# Therapeutic strategies for EGFR-mutated non-small cell lung cancer patients with osimertinib resistance

**DOI:** 10.1186/s13045-022-01391-4

**Published:** 2022-12-08

**Authors:** Kai Fu, Fachao Xie, Fang Wang, Liwu Fu

**Affiliations:** State Key Laboratory of Oncology in South China, Collaborative Innovation Center for Cancer Medicine, Guangdong Esophageal Cancer Institute, Sun Yat-sen University Cancer Center, Guangzhou, 510060 People’s Republic of China

**Keywords:** Osimertinib, EGFR-TKIs, NSCLC, Resistance mechanisms, Therapeutic strategy, Targeted therapy, Chemotherapy, Immunotherapy, Precise medicine

## Abstract

Epidermal growth factor receptor tyrosine kinase inhibitors (EGFR-TKIs) are the preferential options for advanced non-small cell lung cancer (NSCLC) patients harboring EGFR mutations. Osimertinib is a potent irreversible third-generation EGFR-TKI targeting EGFR mutations but has little effect on wild-type EGFR. In view of its remarkable efficacy and manageable safety, osimertinib was recommended as the standard first-line treatment for advanced or metastatic NSCLC patients with EGFR mutations. However, as the other EGFR-TKIs, osimertinib will inevitably develop acquired resistance, which limits its efficacy on the treatment of EGFR-mutated NSCLC patients. The etiology of triggering osimertinib resistance is complex including EGFR-dependent and EGFR-independent pathways, and different therapeutic strategies for the NSCLC patients with osimertinib resistance have been developed. Herein, we comprehensively summarized the resistance mechanisms of osimertinib and discuss in detail the potential therapeutic strategies for EGFR-mutated NSCLC patients suffering osimertinib resistance for the sake of the improvement of survival and further achievement of precise medicine.

## Introduction

Lung carcinoma constitutes the major cause of cancer-related mortality and has the second greatest prevalence worldwide, according to a recent report [1]. Non-small cell lung cancer (NSCLC), which comprises adenocarcinoma and squamous cell carcinoma, is the most frequent histological subtype of lung cancer, accounting for approximately 85% of all patients [2]. Generally, surgical resection is considered the standard procedure for early-stage NSCLC, while chemotherapy is widely applied in advanced NSCLC patients. In particular, molecular targeted therapy regimens are routinely recommended to treat NSCLC patients harboring corresponding genetic mutations [3, 4]. Epidermal growth factor receptor tyrosine kinase inhibitors (EGFR-TKIs) are given priority for those with EGFR-sensitizing mutations, including EGFR exon 19 deletion (Ex19del) and L858R mutations [5, 6]. Since EGFR-TKIs were developed and subsequently applied in the clinic, the survival and clinical outcomes of EGFR-mutated NSCLC patients have remarkably improved, and as a consequence, EGFR-TKIs are recommended as the standard first-line treatment in NSCLC patients with EGFR mutations [7, 8].

There are currently three generations of EGFR-TKIs approved to treat NSCLC patients bearing EGFR mutations in different clinical settings. First-generation (gefitinib, erlotinib and icotinib) and second-generation (afatinib and dacomitinib) EGFR-TKIs have demonstrated substantial clinical benefit in advanced NSCLC patients with Ex19del and L858R mutations [7, 8]. Despite initial potent responses to first- and second-generation EGFR-TKIs (both called early-generation EGFR-TKIs below), a large number of patients will develop acquired resistance within 9 to 14 months during or after EGFR-TKI therapy [9]. T790M mutation, produced by the substitution of threonine to methionine at amino acid position 790 in exon 20 of the EGFR gene, confers resistance to patients receiving early-generation EGFR-TKIs and constitutes the most frequent resistance mechanism, with a proportion of 50%-60% in such a population [10–12]. The emergence of the T790M mutation hampers the binding of early-generation EGFR-TKIs to the ATP-binding site of EGFR, therefore reducing the inhibition of the downstream signaling pathway mediated by EGFR-TKIs and contributing to the development of resistance [13]. Fortunately, the issue of decreased sensitivity to early-generation EGFR-TKIs elicited by the T790M resistance mutation has been solved with the development of third-generation EGFR-TKIs, particularly osimertinib.

Osimertinib is a novel oral, irreversible third-generation EGFR-TKI that targets EGFR-sensitizing and T790M resistance mutations [14, 15]. Osimertinib irreversibly and specifically binds to the EGFR tyrosine kinase domain in a covalent bond formation manner by targeting the cysteine at position 797 in the ATP-binding site [16, 17]. Importantly, osimertinib is almost 200-fold more potent against the T790M mutation than its wild-type counterpart, which minimizes the skin and gastrointestinal toxicity caused by wild-type EGFR inhibition [18]. Clinical data from AURA serial studies suggested that osimertinib was an effective regimen with a manageable safety profile for EGFR-mutant, T790M-positive advanced NSCLC patients. Because of the encouraging results from these clinical trials, osimertinib became the first third-generation EGFR-TKI to receive approval for the clinical application for NSCLC patients with EGFR T790M mutation [19–21]. More importantly, in view of the FLAURA study, osimertinib was recommended as the first-line treatment of advanced NSCLC patients possessing EGFR-activating mutations, regardless of T790M status [22–25]. However, similar to early-generation EGFR-TKIs, patients receiving osimertinib inevitably experience acquired resistance, and multiple mechanisms encompassing EGFR-dependent and EGFR-independent resistance mechanisms have been identified [26–29]. The advent of osimertinib resistance is a major challenge to the successful long-lasting treatment of EGFR-mutated NSCLC patients. Here, we outlined the resistance mechanisms of osimertinib and discussed in detail the corresponding therapeutic strategies for EGFR-mutated NSCLC patients suffering osimertinib resistance.

## Development of three generations of EGFR-TKIs

**Fig. 1:**
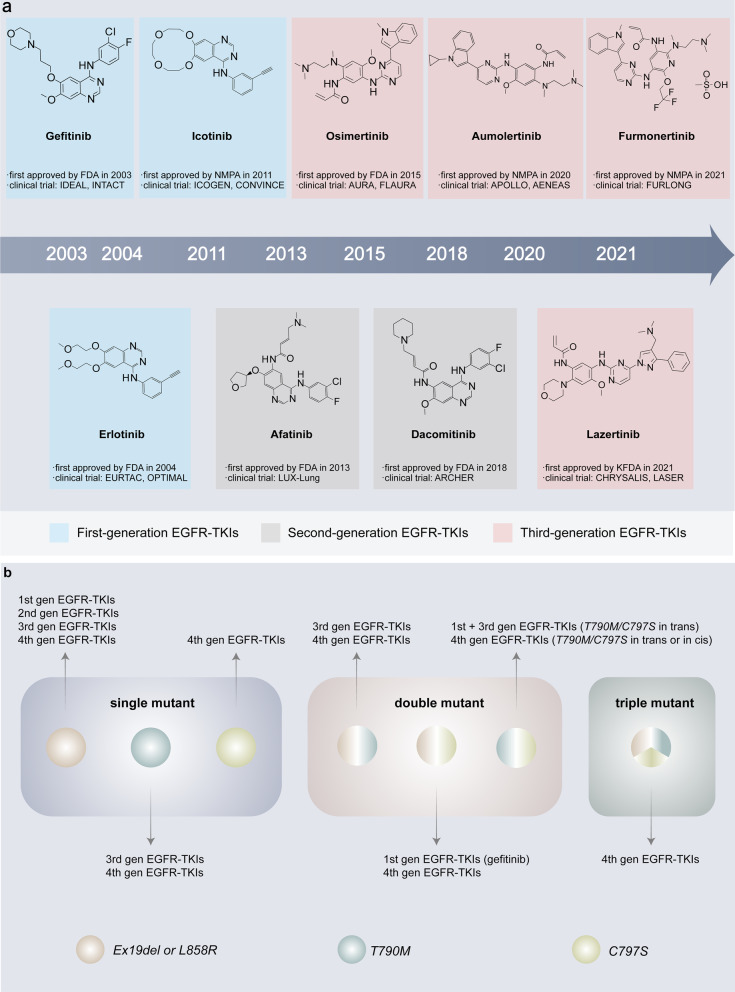
The development of EGFR-TKIs. **a** Timeline of approval of all three generations of EGFR-TKIs in EGFR-mutated NSCLC**.** The chemical structures and the first approval time of EGFR-TKIs are presented. **b** The coverage of common EGFR mutations of four generations of EGFR-TKIs. EGFR mutations are comprised of single mutant, double mutant and triple mutant, which are targeted by corresponding EGFR-TKIs. FDA, US Food and Drug Administration; NMPA, China National Medical Products Administration; KFDA, Korea Food and Drug Administration

Over the past few decades, with the in-depth study of tumor biology, an increasing number of molecular targets for drug therapy have been explored, including EGFR. EGFR is a member of the erbB family of cell surface receptors, which have an extracellular ligand-binding domain, a hydrophobic transmembrane domain and an intracellular tyrosine kinase domain. When a ligand binds to the extracellular domain of EGFR, EGFR dimerizes and activates tyrosine kinase activity in the intracellular domain, giving rise to phosphorylation of intracellular molecules, which leads to a series of activation of signaling pathways implicated in proliferation, invasion, metastasis, etc. [30]. EGFR was discovered in 1977, and EGFR mutations were found to predict sensitivity to gefitinib among NSCLC patients at the beginning of the twenty-first century, which showed encouraging efficacy in EGFR-mutated NSCLC in clinical trials, indicating that the treatment of NSCLC has entered the era of targeted therapy [31, 32]. Since the first EGFR-TKI was approved by the FDA in 2003 for the treatment of advanced NSCLC patients whose diseases progressed after platinum plus paclitaxel chemotherapy, the development of EGFR-TKIs has achieved remarkable success, and there have been three generations of EGFR-TKIs used in clinic (Table 1, Fig. 1).

**Table 1:** Comparison of three-generation EGFR-TKIs

Representative drugs	Types of inhibitors	Targets of EGFR	ORR (%)	PFS (months)	OS (months)	Ref
First-generation EGFR- TKIs						
Gefitinib	Reversible	Ex19del, L858R	67–74	9.2–11.9	30.5–38.8	[5, 6]
Erlotinib	Reversible	Ex19del, L858R	64	9.7–13.1	19.3	[34, 35]
Icotinib	Reversible	Ex19del, L858R	/	11.2	30.5	[36, 37]
Second-generation EGFR-TKIs						
Afatinib	Irreversible	Ex19del, L858R	70	11.0–13.6	19.6–27.6* 30.7–33.3*	[7, 39, 41, 42]
Dacomitinib	Irreversible	Ex19del, L858R	75	14.7	34.1	[8, 44]
Third-generation EGFR- TKIs						
Osimertinib	Irreversible	Ex19del, L858R T790M	80	18.9	38.6	[22, 46]
Aumolertinib	Irreversible	Ex19del, L858R T790M	74	19.3	NR	[47]
Furmonertinib	Irreversible	Ex19del, L858R T790M	74	20.8	NR	[48, 243]
Lazertinib	Irreversible	Ex19del, L858R T790M	55	11.1	NR	[244, 245]

*EGFR-TKIs* epidermal growth factor receptor tyrosine kinase inhibitors, *wt* wild type, *ORR* objective response rate, *PFS* progression-free survival, *OS* overall survival, *NR* not reached

*The upper data was derived from patients with L858R and the lower data was derived from patients with Ex19del. All exhibited data were derived from clinical trials in which drug as monotherapy in the first-line setting

### First-generation EGFR-TKIs

Gefitinib, the first EGFR-TKI, was designed to inhibit the tyrosine kinase activity of EGFR by competitively binding to the ATP-binding site at the intracellular domain of the receptor [33]. After it was indicated that NSCLC with Ex19del and L858R was highly responsive to gefitinib, several randomized phase 3 trials were initiated to compare gefitinib with standard platinum doublet chemotherapy in chemotherapy-naive patients. It was confirmed that the gefitinib group had significantly longer progression-free survival (PFS) than the standard chemotherapy group, with a median PFS of 9.2 months versus 6.3 months and 10.8 months versus 5.4 months, respectively [5, 6]. On account of these studies, gefitinib gained access to approval for the first-line treatment of the NSCLC population harboring EGFR mutations in 2010. Besides gefitinib, two other first-generation EGFR-TKIs, erlotinib and icotinib, were also developed, and all three drugs are reversible, ATP-competitive inhibitors of EGFR. The safety and efficacy of erlotinib compared with standard chemotherapy in the first-line treatment of Asian and European patients with advanced EGFR mutation-positive NSCLC were assessed in the OPTIMAL and EURTAC trials, respectively [34, 35]. The OPTIMAL trial demonstrated that the median PFS was 13.1 months in erlotinib-treated patients and 4.6 months in the chemotherapy group, and the EURTAC study showed a median PFS of 9.7 versus 5.2 months in the erlotinib group and standard chemotherapy group. Icotinib is the first EGFR-TKI to be developed by Chinese researchers. The ICOGEN trial demonstrated that icotinib was noninferior to gefitinib in terms of PFS (4.6 months vs. 3.4 months) in previously treated, nonselected advanced NSCLC patients, and this study fostered the approval of icotinib for NSCLC by the National Medical Products Administration (NMPA) in China [36]. The phase III CONVINCE trial aimed to assess the efficacy and safety of first-line icotinib versus chemotherapy in lung adenocarcinoma patients with exon 19/21 EGFR mutations [37]. The final results showed significantly longer PFS (11.2 versus 7.9 months; *P* = 0.006) and fewer treatment-related adverse events (79.1% versus 94.2%; *P* < 0.001) in the icotinib group than in the chemotherapy group, indicating that icotinib could be considered a first-line treatment for this patient population. The three first-generation EGFR-TKIs all presented superior efficacy and safer profiles as front-line treatments compared with standard chemotherapy in EGFR-mutated NSCLC patients. Nevertheless, a large number of patients will develop acquired resistance within 9 to 14 months during or after EGFR-TKI therapy, with the T790M mutation being the most common resistance mutation [9]. In addition, rash and diarrhea are common adverse events due to inhibition of wild-type EGFR by first-generation EGFR-TKIs. Notably, interstitial lung disease is a relatively rare but severe adverse event associated with gefitinib treatment, which should attract the attention of physicians [38].

### Second-generation EGFR-TKIs

To solve the issues of acquired resistance to first-generation EGFR-TKIs, second-generation EGFR-TKIs have been exploited. Compared to reversible first-generation EGFR-TKIs, afatinib and dacomitinib irreversibly inhibit the tyrosine kinase domain of EGFR. Afatinib is a pan-ErbB family blocker with preclinical activity against T790M, which irreversibly blocks signals from EGFR/ErbB1, HER2/ErbB2 and ErbB4. The LUX-Lung 3 and LUX-Lung 6 clinical trials compared afatinib with cisplatin-based doublet chemotherapy in advanced NSCLC patients with EGFR mutation [39, 40]. Both trials demonstrated that first-line afatinib significantly improved PFS (13.6 months vs. 6.9 months and 11.0 months vs. 5.6 months, respectively). Although there was no difference in overall survival (OS) between the afatinib group and the chemotherapy group in the whole population of two trials, improved OS was observed in patients with Ex19del who were administered afatinib, suggesting that Ex19del-positive NSCLC might be more sensitive to afatinib than L858R-positive disease [41]. The LUX-Lung 7 study further compared the efficacy and safety of afatinib and gefitinib in the first-line treatment of EGFR mutation-positive NSCLC [7, 42]. Afatinib prolonged the time to treatment failure (13.7 months vs. 11.5 months), but no significant differences were observed in PFS or OS (11.0 months vs. 10.9 months and 27.9 vs. 24.5 months, respectively). In terms of safety, the most common treatment-related grade 3 or 4 adverse events were diarrhea (13% in the afatinib group vs. 1% in the gefitinib group) and rash (9% in the afatinib group vs. 3% in the gefitinib group). Furthermore, 17 (11%) patients given afatinib and 7 (4%) patients given gefitinib were subjected to serious treatment-related adverse events. The higher frequency of afatinib treatment-related adverse events was perhaps ascribed to the wide spectrum of inhibition of the pan-ErbB family. It is noteworthy that although afatinib presented preclinical activity against T790M, no clinical efficacy was observed in NSCLC patients with the T790M mutation. Another irreversible pan-ErbB family TKI, dacomitinib, was not superior to erlotinib in an unselected, previously treated, advanced NSCLC patient population in the ARCHER 1009 study, whereas in the ARCHER 1050 trial, dacomitinib significantly improved PFS (14.7 months vs. 9.2 months) and OS (34.1 months vs. 26.8 months) over gefitinib in the first-line setting [8, 43, 44]. Similar to afatinib, the prevalence of treatment-related serious adverse events was higher in patients given dacomitinib than gefitinib.

Overall, early-generation EGFR-TKIs markedly improve the survival and clinical outcomes of EGFR-mutated NSCLC patients and have a manageable safety profile compared with chemotherapy. However, the short PFS (only about 10 months), high incidence of adverse events such as rash and diarrhea because of the inhibition of wild-type EGFR, and resistance limit successful therapy of NSCLC patients in clinic. To address these issues, third-generation EGFR-TKIs were expected to develop.

### Third-generation EGFR-TKIs

Osimertinib is the first third-generation EGFR-TKI approved by the FDA and EMA for the treatment of metastatic NSCLC patients with the EGFR T790M mutation [21], which irreversibly binds to the cysteine at position 797 (C797) in the ATP-binding site of the EGFR tyrosine kinase domain in a covalent bond formation manner. Osimertinib was designed to selectively target both EGFR-sensitizing mutations and the T790M resistance mutation while sparing wild-type EGFR. Furthermore, the ability of osimertinib to penetrate the blood‒brain barrier (BBB) is more potent than that of other EGFR-TKIs. These characteristics confer osimertinib with higher selectivity and lower toxicity. Importantly, osimertinib overcomes acquired resistance to early-generation EGFR-TKIs induced by T790M and shows potential efficacy against central nervous system (CNS) metastases. AURA serial clinical trials assessed the efficacy and safety of osimertinib compared with standard chemotherapy or first-generation EGFR-TKIs (Table 2). The AURA3 study showed that osimertinib had significantly longer PFS (10.1 months vs. 4.4 months) than pemetrexed plus platinum in patients with T790M-positive advanced NSCLC in the second-line setting, even in patients with brain metastasis (8.5 months vs. 4.1 months) [45]. And then, the FLAURA trial demonstrated that osimertinib exhibited superior efficacy compared to gefitinib or erlotinib in the first-line treatment in terms of PFS (18.9 months vs. 10.2 months) and OS (38.6 months vs. 31.8 months), with lower rates of serious adverse events [22, 46]. Notably, osimertinib also prolonged PFS in patients with CNS metastases (15.2 months vs. 9.6 months) in the first-line setting. As a consequence, osimertinib was recommended to become first-line therapy in advanced or metastatic EGFR mutant-positive NSCLC on the strength of the FLAURA study, regardless of T790M status. Another two irreversible third-generation EGFR-TKIs, aumolertinib (almonertinib, HS-10296) and furmonertinib (alflutinib, AST2818), were developed on the basis of osimertinib. Compared with gefitinib, aumolertinib and furmonertinib both also had longer median PFS (19.3 months vs. 9.9 months and 20.8 months vs. 11.1 months, respectively) as the first-line treatment for EGFR-mutated NSCLC in the AENEAS and FURLONG phase 3 studies, with an acceptable safety profile [47, 48]. They were approved by NMPA in China for the treatment of patients harboring T790M on March 18, 2020, and March 3, 2021, respectively [49, 50]. Finally, lazertinib received its first approval from the Korea Food and Drug Administration (KFDA) for the treatment of locally advanced or metastatic NSCLC patients with the T790M mutation who were pretreated with EGFR-TKIs in January 2021 [51].

**Table 2:** Efficacy and safety of osimertinib in clinical trials

Trial	Phase	Number of patients	ORR (%)	PFS (months)	OS (months)	AEs of osimertinib treatment
AURA (NCT01802632)	1/2	253	61	9.6	/	Diarrhea (47%), rash (40%), nausea (22%), decreased appetite (21%)
AURA extension	2	201	62	12.3	/	Diarrhea (43%), rash (40%), dry skin (31%), paronychia (31%)
AURA2 (NCT02094261)	2	210	70	9.9	/	Rash (41%), diarrhea (33%), dry skin (30%), paronychia (26%)
AURA extension and AURA2	2	411	66	9.9	26.8	Rash (42%), diarrhea (39%), dry skin (32%), paronychia (32%)
AURA3 (NCT02151981)	3	279	71	10.1	26.8	Diarrhea (41%), rash (34%), dry skin (23%), paronychia (22%)
FLAURA (NCT02296125)	3	279	80	18.9	38.6	Rash (58%), diarrhea (58%), dry skin (36%), paronychia (35%)
ADAURA (NCT02511106)	3	682	57.1	NR* (38.8-NC)	Awaited	Diarrhea (46%), paronychia (25%), dry skin (23%), pruritus (19%)

*ORR* objective response rate, *PFS* progression-free survival, *OS* overall survival, *AEs* adverse events, *NR* not reached, *NC* could not be calculated

*represents disease-free survival (DFS)

In summary, the rapid development of EGFR-TKIs has revolutionized the treatment patterns of EGFR-mutated NSCLC, which improved the survival and quality of life of this population. Nevertheless, similar to early-generation EGFR-TKIs, intractable matters of acquired resistance to osimertinib also inevitably occur, posing challenges to the long-term effective management of NSCLC patients. The in-depth investigation of the exact resistance mechanisms of osimertinib sheds light on the exploration of corresponding therapeutic strategies for the sake of improving the clinical outcomes of the NSCLC population. The underlying mechanisms of resistance to osimertinib and current feasible therapeutic strategies will be discussed in the following sections.

## Overview of resistance mechanisms to osimertinib

**Fig. 2:**
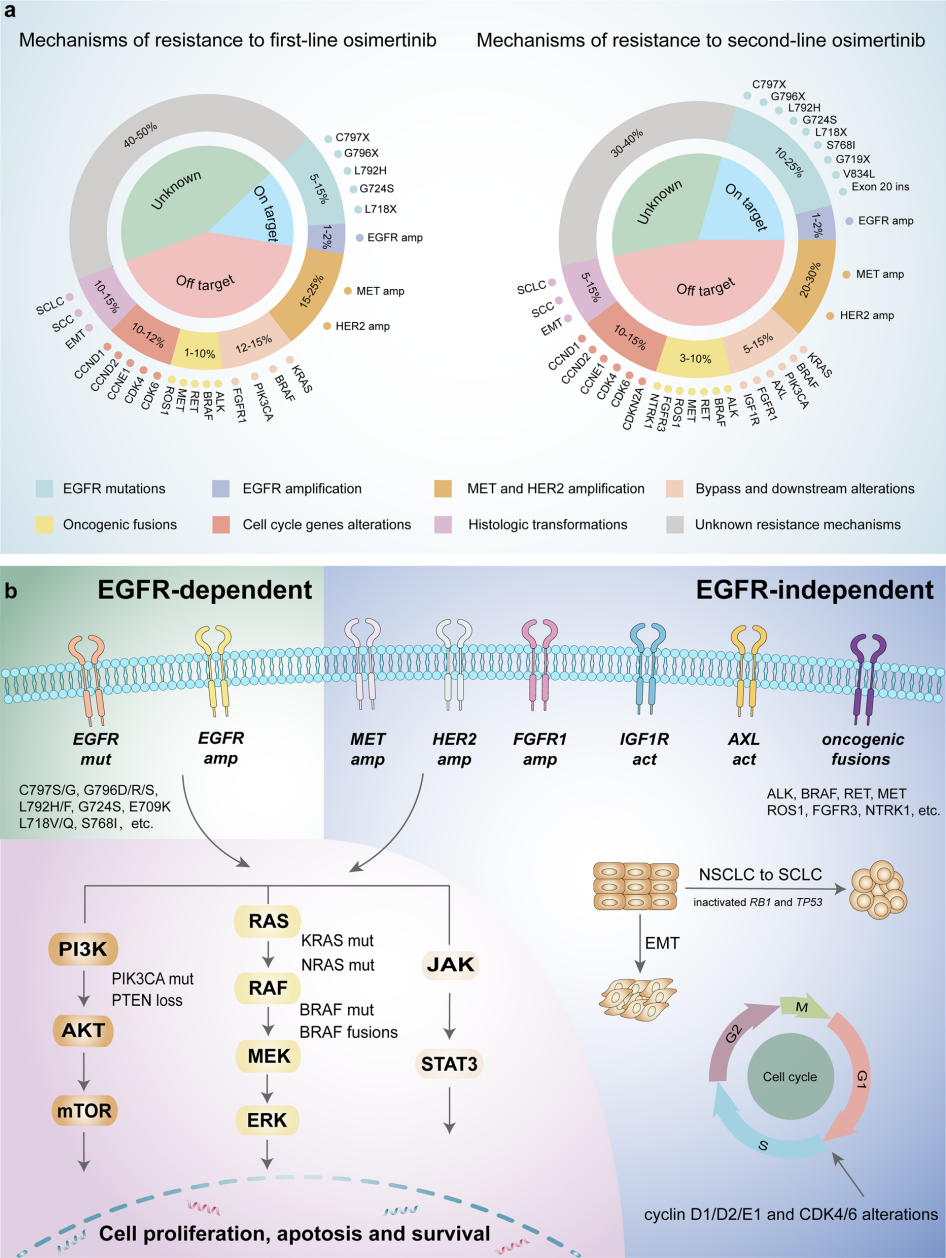
Mechanisms of resistance to osimertinib. **a** Resistance mechanisms of osimertinib occur in first-line (left) and second-line therapy (right), and incidences of each class of resistance mechanisms are also presented. **b** Resistance mechanisms to osimertinib are broadly divided into EGFR-dependent and EGFR-independent resistance mechanisms. The latter include bypass signaling activation, oncogenic fusions, downstream pathway alterations, histologic transformations and cell cycle gene alterations. Abbreviations: act, activation; amp, amplification; mut, mutation

EGFR-TKIs have dramatically improved the clinical outcomes of NSCLC patients harboring EGFR mutations. Nevertheless, despite the longer period of survival and lower rates of adverse events associated with EGFR-TKIs, a majority of patients eventually presented resistance to EGFR-TKIs, regardless of the lines of treatment. Various acquired resistance mechanisms are broadly divided into EGFR-dependent (on-target) and EGFR-independent (off-target) resistance mechanisms (Fig. 2) [52–56]. The relative incidence of on-target and off-target resistance mechanisms differs between early-generation EGFR-TKIs and third-generation EGFR-TKIs. Patients predominantly develop on-target resistance mechanisms when they progress on early-generation EGFR-TKIs, with T790M accounting for approximately 50% of cases [9]. However, on-target resistance occurs in only 10–20% of patients treated with osimertinib, and C797S mutation represents the most common on-target resistance mechanism [57, 58]. The lower incidence of on-target resistance to osimertinib possibly reflects better on-target inhibition and discrepancy in selective pressure and/or clonal evolution of EGFR-mutated NSCLC compared with early-generation EGFR-TKIs [59].

### EGFR-dependent resistance mechanisms

On-target resistance mechanisms mainly involve alterations in critical amino acid residues that sterically interfere with the binding of osimertinib to the ATP-binding site of the EGFR tyrosine kinase domain, thus resulting in drug resistance.

#### C797X mutations

The cysteine residue at position 797 (C797) in exon 20 of EGFR is the covalent binding site of irreversible osimertinib, and point mutations at C797 (C797X) interfere with drug-protein interaction. C797X mutations (mainly C797S) have become the most frequent EGFR-dependent resistance mechanism to osimertinib, with different incidences depending on the treatment setting. The incidence of C797X mutations is 15% at disease progression on second-line osimertinib in the AURA3 trial, whereas they were detected in only 7% of patients in the first-line setting of the FLAURA trial at the time of disease progression [57, 58]. In other studies, the prevalence of C797X mutations ranges from 0 to 29%, at least in part reflecting intratumor heterogeneity [56, 60–66].

#### Other EGFR-dependent resistance mechanisms

Mutations at other amino acid residues of EGFR have also been confirmed to confer resistance to osimertinib. G796 is located under the phenyl aromatic ring of osimertinib, where solvent-front resistant mutations occur and G796X mutations (G796D, G796R and G796S) sterically hamper the binding of osimertinib to the EGFR intracellular kinase domain [60, 66–68]. Similarly, L792 has a close interaction with the methoxy group on the aromatic ring of osimertinib, generating steric hindrance to osimertinib [56, 66]. Interestingly, L792 mutations usually coexist with other EGFR mutations and were always in cis with T790M but in trans with G796/C797 alterations, suggesting that L792 mutations may independently lead to osimertinib resistance [69]. Furthermore, L718 forms a “hydrophobic sandwich” with the phenyl aromatic ring of osimertinib, at which mutations interfere with drug-protein binding, conferring resistance to osimertinib [70, 71]. Therefore, L718Q, L718V and L792H all prevent osimertinib from the binding to EGFR by spatial conflict [66, 72]. Structural analyses and computational modeling indicated that the G724S mutation may induce a conformational change in the kinase domain that is incompatible with the binding of third-generation TKIs [73, 74].

EGFR amplification is another EGFR-dependent resistance mechanism to EGFR-TKIs. Amplification of wild-type EGFR contributes to acquired resistance to osimertinib through EGFR ligand-induced activation, which might possibly be due to the decreased affinity to wild-type EGFR by mutation-selective osimertinib [75]. Interestingly, we found wild-type EGFR could transfer to sensitive cells with EGFR mutation via exosomes, which result in cell resistance to osimertinib [76].

It is worth noting that approximately 50% of EGFR T790M-positive NSCLC patients at baseline are likely to suffer loss of T790M after osimertinib treatment in certain studies. [56, 62, 63]. Importantly, a variety of alternative resistance mechanisms (e.g., MET amplification, gene fusions, SCLC transformation) were identified in the case of T790M loss [63, 77]. This phenomenon was potentially associated with intratumor heterogeneity, selective pressure and clonal evolution, resulting in the diversity of resistance mechanisms in EGFR-mutated NSCLC with osimertinib resistance. Therefore, these suggested that the NSCLC patients with the loss of T790M may not be suitable to re-treat with early-generation EGFR-TKIs, which is attributable to the emergence of novel competing off-target resistance mechanisms.

### EGFR-independent resistance mechanisms

Compared with EGFR-dependent mechanisms, EGFR-independent mechanisms are more significant and frequent in osimertinib resistance, perhaps owing to better on-target inhibition of osimertinib.

#### MET amplification

The MET oncogene encodes the receptor tyrosine kinase c-Met, resulting in phosphorylation of the receptor and activation of bypass of EGFR downstream signaling pathways (e.g., PI3K/AKT, JAK/STAT3, RAS/MAPK/ERK) by binding to the ligand HGF. The MET signaling pathway plays an important role in cell migration, apoptosis, and proliferation and promotes tumor angiogenesis, invasion and metastasis [78]. MET amplification has long been considered an important off-target resistance mechanism to early-generation EGFR-TKIs, which is now also identified as the most frequent EGFR-independent resistance mechanism of osimertinib, accounting for 5–24% of patients with progression on osimertinib [9, 57, 58, 61, 63]. Intriguingly, a retrospective study analyzed possible resistance mechanisms of osimertinib as the first- and second-line therapy utilizing next-generation sequencing (NSG)-based liquid biopsy and found that first-line osimertinib resistance commonly included MET amplification, whereas C797S was more common in the second-line setting, which was consistent with previous reports [57, 58, 79].

#### HER2 amplification

HER2 (ErbB2) and EGFR (ErbB1) belong to the ErbB receptor tyrosine kinase family, and both share common downstream signaling pathways. HER2 directly activates EGFR downstream signaling to mediate osimertinib resistance. Paired plasma samples collected at baseline and disease progression identified that HER2 amplification was detected in 2–5% of patients in AURA3 and FLAURA clinical trials [57, 58]. Another HER2 alteration, exon 16-skipping HER2, contributes to osimertinib resistance via a Src-independent pathway through the formation of the HER2 splice variant HER2D16 [80].

#### Alterations in other tyrosine kinase receptors

AXL is a member of the receptor tyrosine kinase family, which is involved in tumor growth, invasion and metastasis. High expression of AXL was reported to be associated with poor prognosis in several types of cancer, including NSCLC. Compared to those with wild-type EGFR, AXL was overexpressed more frequently in lung adenocarcinomas harboring EGFR-activating mutations [81]. Activated AXL could maintain cell survival and induce resistance to osimertinib by interacting with EGFR and HER3 [82]. In addition, fibroblast growth factor receptor 1 (FGFR 1) amplification and activation of insulin-like growth factor 1 receptor (IGF1R) have been reported to confer osimertinib resistance [83, 84].

#### Oncogenic fusions

Oncogenic fusions are responsible for acquired resistance to osimertinib as oncogenic drivers that activate bypass signaling pathways, which are observed in 1%-10% of cases [64]. Oncogenic fusions involving RET, MET, BRAF, ALK, FGFR3, and NTRK1 were confirmed in the MATCH-R study [61]. Particularly, CCDC6–RET, CAV1-MET, AGK-BRAF, EML4-ALK, FGFR3-TACC3, TPM3–NTRK1, GOPC-ROS1, etc. were identified to mediate osimertinib resistance [85–90].

#### Alterations in signaling pathways

EGFR exerts biological functions by activating downstream signaling pathways (e.g., PI3K/AKT, RAS/MAPK/ERK, JAK/STAT) upon binding its ligand. Alterations in these signaling pathways have also been reported to contribute to resistance to osimertinib. RAS and RAF are upstream genes of MAPK signaling, and RAS/RAF mutations including KRAS, NRAS and BRAF mutations conferred resistance to osimertinib. KRAS mutations were detected in 1–7% of patients with osimertinib resistance, while BRAF mutations (mainly BRAF V600E) were detected in 3–4% of cases [57, 58, 63, 66, 91, 92]. PIK3CA encodes the p110α protein, the catalytic subunit of PI3K, which is implicated in regulating the PI3K/AKT pathway. PIK3CA mutations contribute to persistent activation of the PI3K/AKT pathway, which promotes tumorigenesis, proliferation, migration, invasion and resistance to therapy. PIK3CA mutations were often observed in patients progressing on osimertinib therapy, with an incidence of 17% revealed by Lee et al. [56–58, 65, 66]. In addition, loss of PTEN is responsible for increased activation of PI3K signaling, which has also been identified as a resistance mechanism of osimertinib [93].

#### Cell cycle gene alterations

Cell cycle gene alterations have been found in 10–12% of patients who progressed on the therapy of osimertinib and have been reported to be associated with shorter median PFS (4.4 vs. 8.8 months) [56–58, 65]. Alterations in cell cycle-related genes involve cyclin D1, D2 and E1 genes, cyclin-dependent kinase (CDK) 4 and 6 genes and the CDK inhibitor 2A gene (CDKN2A) [55, 57, 58].

#### Histologic transformations

Histologic transformation from NSCLC to squamous cell carcinoma (SCC) or small cell lung cancer (SCLC) has been identified in 2–15% of patients subjected to progression on osimertinib treatment [60, 63, 94]. Complete inactivation of both RB1 and TP53 was strikingly associated with an increased risk of transformation from NSCLC to SCLC among EGFR-mutant lung adenocarcinomas, suggesting the predictive value of these two mutations in SCLC transformation [95, 96]. Although concurrent RB1 and TP53 alterations do not represent small cell transformation, it is necessary to determine histologic type using tissue biopsy in the cases of the emergence of inactivated RB1 and TP53. In addition, epithelial-mesenchymal transition (EMT), another type of histologic transformation, has also been described as a mechanism of osimertinib resistance, which is related to NF-κB and TGFβ2 [56, 97].

## Therapeutic strategies for osimertinib resistance

**Fig. 3:**
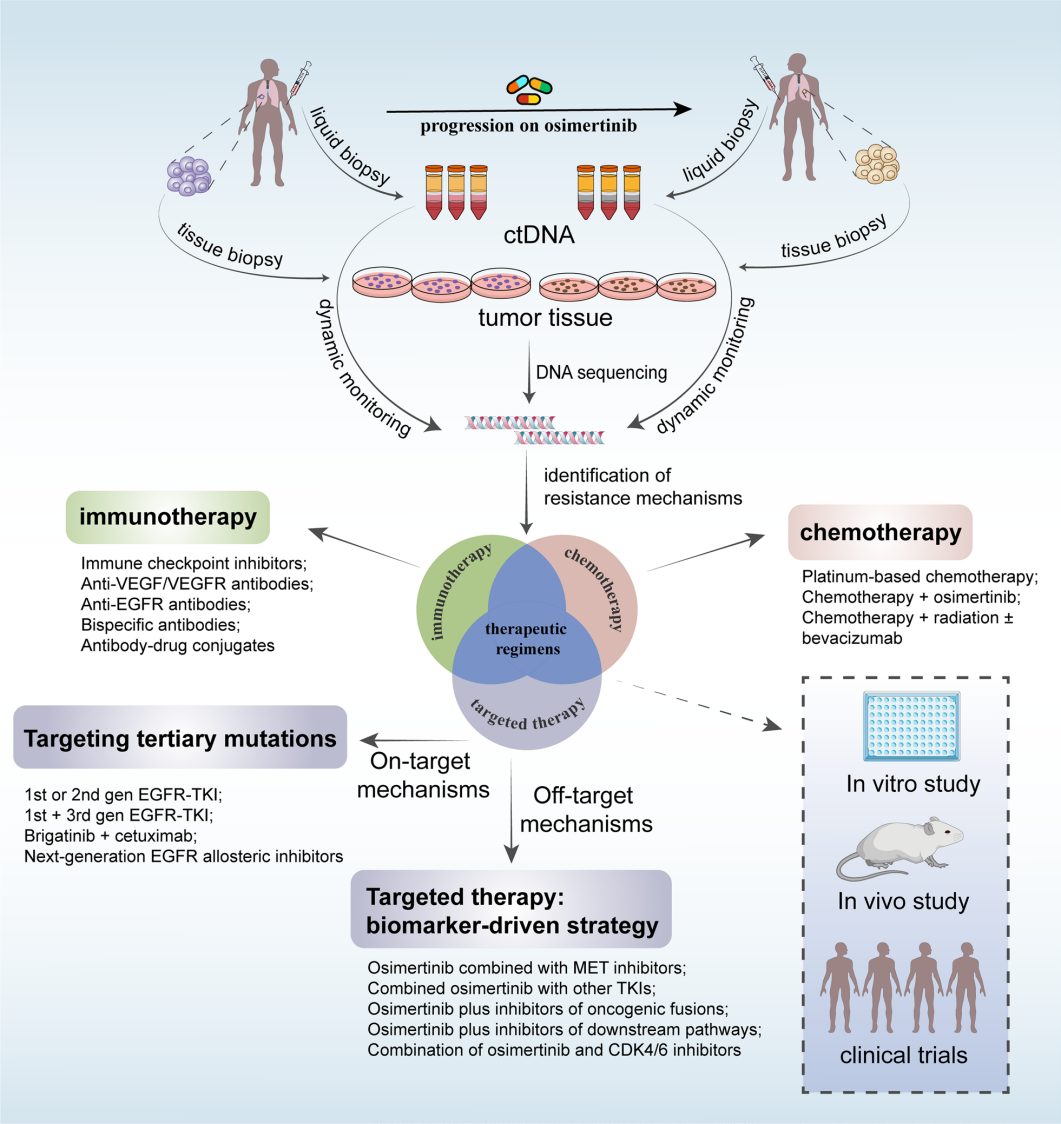
The procedures after osimertinib resistance. Before osimertinib treatment, patients should be examined via tissue biopsy and/or liquid biopsy to confirm whether EGFR mutations exist. Liquid biopsy can be used as a noninvasive and practical method for dynamic monitoring to evaluate genomic status in the period of osimertinib treatment. When patients progress on osimertinib, tissue biopsy and/or liquid biopsy should be conducted to determine the mechanisms of resistance to osimertinib to guide treatment. A corresponding treatment regimen will be applied to treat patients if effective approaches currently exist; in the case of a lack of valid targets, preclinical studies and clinical trials are warranted to be taken into consideration for the acquisition of valuable evidence to further explore viable therapeutic strategies

The spatiotemporal heterogeneity of tumor facilitates the complexity and diversity of molecular resistance mechanisms. Moreover, the coexistence of several resistance mechanisms and constant changes in resistance mechanisms enhance the difficulty in developing effective therapeutic strategies and lead to poor responses. The emergence of osimertinib resistance imposes restrictions on successful long-term treatment and poses a tremendous challenge to EGFR-mutant NSCLC patients, regardless of T790M status. A comprehensive understanding of the mechanisms of resistance to osimertinib contributes to determining the matters of osimertinib resistance and further exploring therapeutic strategies. Based on the known resistance mechanisms, the potential therapeutic regimens include targeted therapy, platinum-based chemotherapy and immunotherapy. The procedures after osimertinib resistance were shown in Fig. 3.

### Targeted therapy

#### Targeting on-target resistance mechanisms

##### Early-generation EGFR-TKIs

As discussed above, NSCLC patients possibly underwent the loss of T790M and acquired tertiary mutations during osimertinib treatment, and therefore it is seemingly feasible to utilize early-generation EGFR-TKIs to re-treat this group after progression on osimertinib treatment. An in vitro study demonstrated that cells expressing Ex19del/C797S without T790M were resistant to third-generation TKIs but maintained sensitivity to first-generation TKIs [98]. In light of this preclinical evidence, a case report revealed that one patient with Ex19del/C797S achieved a meaningful clinical improvement with gefitinib treatment after resistance to osimertinib [99]. Other less common tertiary EGFR mutations (e.g., G796S, L792H, G724S, and L718X) intervene in the binding of osimertinib to the EGFR kinase domain, conferring resistance to osimertinib. Similarly, the patients with these mutations retain a response to early-generation EGFR-TKIs in the absence of the T790M mutation [100]. A patient with G796S exhibited sensitivity to gefitinib after progression on osimertinib treatment, while dacomitinib showed little efficacy in five EGFR-mutated NSCLC patients with compound mutations, owing to the decreased binding affinity of dacomitinib to EGFR mediated by L792H and L718Q [101, 102]. Additionally, afatinib monotherapy or in combination with osimertinib was used to treat patients with Ex19del/G724S who developed resistance to osimertinib, and both achieved significant effects [103, 104]. For L718X (L718V and L718Q), several case reports have shown improved clinical benefit to the treatment of afatinib in EGFR-mutated NSCLC patients with disease progression on the therapy of osimertinib [72, 105–107]. However, Oxnard et al. thought that in most cases, loss of T790M was related to the emergence of alternative off-target resistance mechanisms, which resulted in the failure of the early-generation EGFR-TKIs treatment [63]. Therefore, readministration of previous EGFR-TKIs may not always be feasible. The complexity of osimertinib resistance in NSCLC patients with loss of T790M highlights the necessity to explore more approaches to prevent or delay the formation of novel mechanisms of resistance to osimertinib.

##### Combination of first- and third-generation EGFR-TKIs

IN the presence of the compound mutations of T790M and C797S, different strategies were developed according to the allelic context of T790M and C797S. Specifically, if C797S and T790M mutations are on different chromosomes (in trans), tumor will retain sensitivity to the combination treatment of first- and third-generation inhibitors; while they are on the same chromosome (in cis), EGFR-TKIs alone or in combination are ineffective [108]. On the basis of preclinical studies, several case reports have demonstrated that the combination of gefitinib/erlotinib and osimertinib was beneficial to EGFR-mutated NSCLC patients harboring T790M in trans with C797S [109–111].

##### Brigatinib plus cetuximab

Considering that EGFR-TKIs alone or combination therapy were ineffective in patients with T790M and C797S in cis, alternative combination treatment regimens have been reported. Brigatinib, a dual ALK and EGFR inhibitor, when combined with cetuximab (anti-EGFR antibody), showed promising efficacy against NSCLC with T790M/cis-C797S EGFR mutations in a preclinical study and a case report, respectively [112, 113]. Furthermore, a retrospective cohort study reported brigatinib plus cetuximab achieved an objective response rate (ORR) of 60% and a median PFS of 14 months, while those who received chemotherapy exhibited an ORR of 10% and a median PFS of 3 months. However, no benefit was observed in another retrospective cohort study of the comparison of brigatinib-based therapy with chemotherapy-based treatment, which may be attributed to the addition of antiangiogenic agents to chemotherapy [114, 115].

Except for the brigatinib-based regimen, other potential regimens targeting T790M-cis-C797S have also been reported. Osimertinib plus anlotinib or afatinib plus apatinib achieved a partial response of 9 months and a PFS of more than 10 months in two case reports, respectively. These suggest that combination of EGFR inhibitors and VEGFR inhibitors is a promising therapy for EGFR-mutated NSCLC patients with T790M in cis with C797S [116, 117]. Recently, an immune checkpoint inhibitor (ICI) combined with platinum-based doublet chemotherapy also exhibited efficacy against T790M-cis-C797S, with a PFS of 8 months, which should be further evaluated in clinical trials [118]. T790M-cis-C797S-mediated resistance to osimertinib has attracted increasing attention; however, at present, no standard therapeutic regimens were recommended for this subset of patients. According to the available limited clinical evidence obtained from retrospective studies, combined therapy with brigatinib and cetuximab may be beneficial, and was recommended for NSCLC patients with T790M in cis with C797S EGFR mutations.

In addition to a few preclinical studies, case reports and retrospective cohort studies, the prospective clinical trials should be carried out to treat the NSCLC patients with tertiary EGFR mutations. The safety and efficacy of these therapeutic strategies should be further assessed in a larger group of this subset of patients. In addition, certain EGFR mutations coexist in the same patient, suggesting a high degree of clonal heterogeneity in the course of osimertinib resistance and disease progression, which poses challenges in the treatment of such a patient population.

##### Next-generation EGFR allosteric inhibitors

As mentioned above, although several potential therapeutic strategies were studied on the NSCLC patients with C797S mutation, the issue of C797S-mediated resistance to osimertinib has not been radically solved. To solve this issue, several next-generation EGFR inhibitors are developed. EGFR allosteric inhibitors bind to EGFR at a site away from the tyrosine kinase domain, which bypasses the C797S-mediated resistance mechanism, showing a promising strategy to overcome osimertinib resistance induced by C797S [119]. EAI045, an EGFR allosteric inhibitor, belongs to the fourth-generation inhibitor that selectively changes the space configuration of mutated EGFR and hinders its binding to EGFR ligands, thus blocks the phosphorylation of itself and its downstream signal pathway, such as p-AKT, p-STATs, and p-ERK1/2. EAI045 plus cetuximab exhibited remarkable synergistic effect on Ba/F3 cells with triple mutants (L858R/T790M/C797S) in vitro and in vivo, due to EGFR dimerization blockade mediated by cetuximab [119, 120]. JBJ-04-125-02 was also an EGFR allosteric inhibitor that could inhibit proliferation of the NSCLC cells with EGFR triple mutants in vitro and in vivo, and combination of JBJ-04-125-02 and osimertinib resulted in increased apoptosis compared with the treatment with JBJ-04-125-02 or osimertinib alone [121]. Compared to JBJ-04-125-02, JBJ-09-063 derived from JBJ-04-125-02 showed more potent efficacy in vitro and in vivo, regardless of whether it was a single agent or in combination with other TKIs [122]. In contrast to EAI045, CH7233163, a promising fourth-generation EGFR-TKI, inhibited growth of NSCLC cells with Del19/T790M/C797S triple EGFR mutants in vitro and in vivo. [123]. Additionally, the in vivo antitumor activity of BLU-701 (a fourth-generation EGFR-TKI) against the C797S resistance mutation has been demonstrated in a PC9 cell line-derived tumor xenograft model [124, 125]. The phase I/II SYMPHONY clinical trial (NCT04862780) is evaluating the safety and anticancer activity of BLU-945 against the EGFR-sensitized mutation/T790M/C797S [126–128]. Recently, circulating tumor DNA (ctDNA) analysis revealed that BLU-945 achieved a median 48% reduction in EGFR resistance mutations (T790M and C797S), which represented emerging evidence of the efficacy of BLU-945 [129]. Finally, other fourth-generation EGFR inhibitors (TQB3804, BBT-176, BBT-207, etc.) are also being investigated [130].

**Fig. 4:**
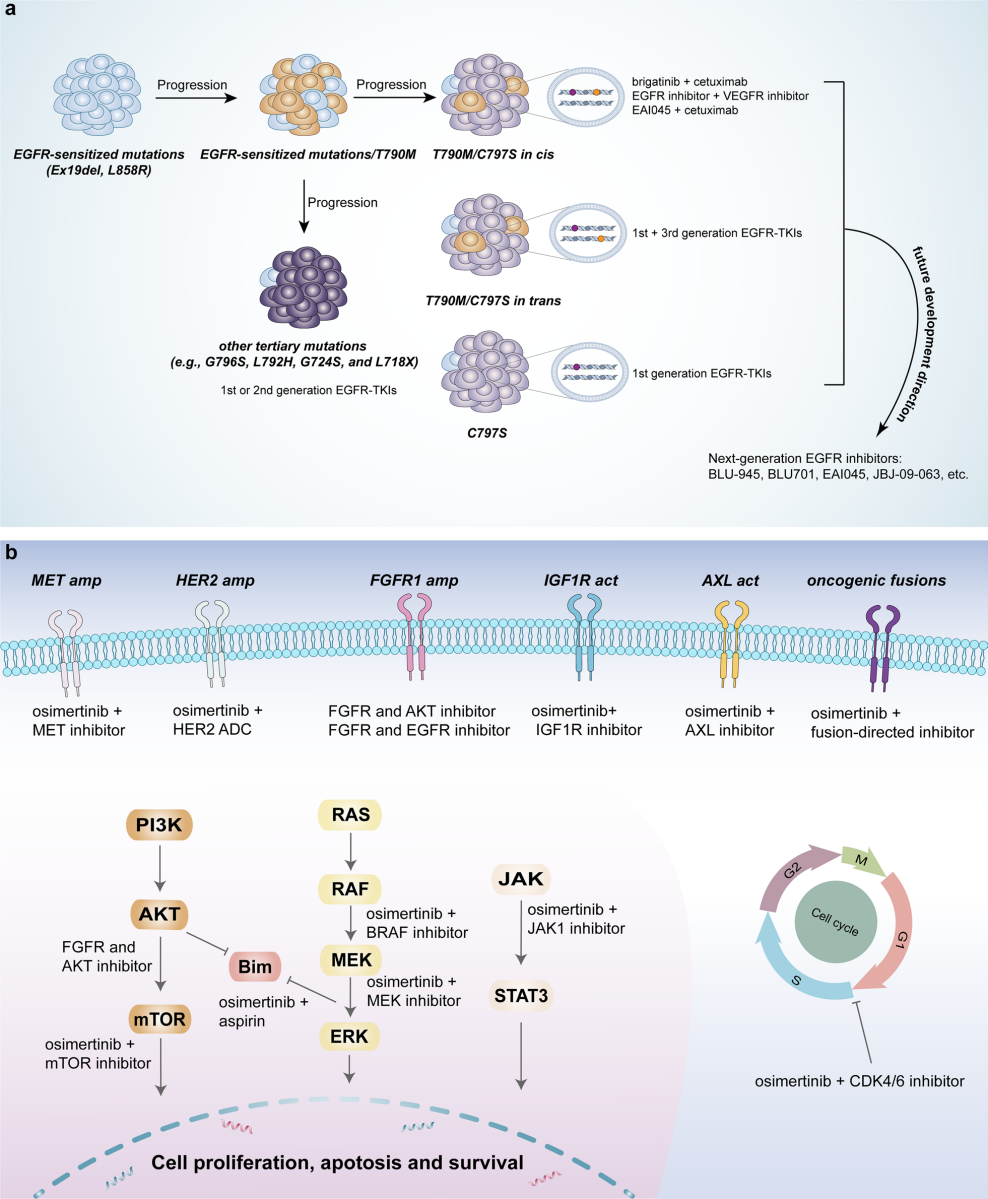
Targeted therapy for osimertinib resistance. **a** Different treatment regimens targeting on-target resistance mechanisms (mainly EGFR tertiary mutations). C797S/T790M in cis: osimertinib-resistant NSCLC responds to brigatinib in combination with cetuximab, and such a regimen has been proven in several case reports. C797S/T790M in trans: NSCLC patients become sensitive to combination therapy of osimertinib and first-generation EGFR-TKIs. C797S alone and the other tertiary EGFR mutations: patients may resensitize to early-generation EGFR-TKIs. Novel EGFR inhibitors are under way to overcome C797S-mediated resistance to osimertinib, regardless of the C797S/T790M allelic context. **b** Targeting common off-target resistance mechanisms. Based on current available preclinical and clinical evidence, combination treatments of osimertinib plus inhibitors of corresponding targets are the major therapeutic strategies.

Although many allosteric inhibitors have shown potent efficacy to inhibit growth of osimertinib-resistant NSCLC cells in preclinical studies, most have not reached the clinical trial stage. To assess the feasibility of allosteric inhibitors in a real-world setting, further clinical studies are warranted to provide insights into the safety and efficacy of such therapeutic strategies. Currently, potential therapeutic strategies targeting tertiary mutations are summarized in Fig. 4a.

#### Targeting off-target resistance mechanisms

##### Osimertinib combined with MET inhibitors

MET amplification has been identified as the most common EGFR-independent mechanism of osimertinib resistance, accounting for 5%-24% of cases [56, 63, 64]. Preliminary clinical evidence has demonstrated that a combination regimen with osimertinib and MET inhibitors is a plausible therapeutic strategy to overcome MET amplification-mediated resistance to osimertinib.

Dual crizotinib and osimertinib treatment showed evident clinical and radiographic responses in EGFR mutation-positive NSCLC patients with MET amplification in case reports [131, 132]. The combined effect of crizotinib and osimertinib was also reported in a retrospective analysis, which showed that the ORR was 100% and the median PFS was 6.2 months in lung adenocarcinoma patients with MET amplification [133]. Importantly, the TATTON study was conducted to evaluate the safety and tolerability of osimertinib combined with selumetinib (MEK1/2 inhibitor), savolitinib (MET inhibitor), or durvalumab (anti-PD-L1 monoclonal antibody) in advanced EGFR-mutant NSCLC patients who progressed on a prior EGFR-TKI [134]. Recently, interim results from TATTON revealed that osimertinib plus savolitinib had acceptable risk–benefit characteristics and encouraging antitumor activity, with an ORR of 64% versus 48% and a median PFS of 9.1 versus 7.6 months in the osimertinib plus savolitinib 300 mg group and osimertinib plus savolitinib 600 mg group, respectively [135]. The ongoing clinical studies (NCT03778229, NCT05015608 and NCT03944772) are also under way to investigate the safety and efficacy of osimertinib combined with savolitinib in patients harboring MET amplification-mediated resistance to osimertinib (Table 3). Moreover, two other MET inhibitors, capmatinib and tepotinib, are being evaluated in combination with osimertinib in two clinical trials, NCT04816214 and NCT03940703, respectively. It is worth mentioning that combination therapy of osimertinib plus capmatinib achieved rapid clinical improvement in a MET-amplified NSCLC patient [136].

**Table 3:** Ongoing trials for EGFR-mutated NSCLC patients with osimertinib resistance

NCT number	Phase	Pts	Treatment arm(s)	Primary endpoint(s)
Targeted therapy				
NCT04862780 (SYMPHONY)	1/2	120	BLU-945 (EGFR inhibitor)	MTD, RP2D, AEs, ORR
NCT03940703 (INSIGHT 2)	2	120	Osimertinib + tepotinib (MET inhibitor)	ORR
NCT04816214	3	245	Osimertinib + capmatinib (MET inhibitor); Platinum + pemetrexed	DLT, PFS
NCT03778229 (SAVANNAH)	2	259	Osimertinib + savolitinib (MET inhibitor)	ORR
NCT05015608 (SACHI)	3	250	Osimertinib + savolitinib; Pemetrexed + platinum	PFS
NCT03255083	1	13	Osimertinib + DS-1205c (AXL inhibitor)	Number of participants with DLT
NCT02503722	1	36	Osimertinib + sapanisertib (mTOR inhibitor)	MTD and DLT
NCT03532698	/	100	Osimertinib and aspirin; Osimertinib	ORR
NCT04001777	1	60	Osimertinib + palcitoclax (Bcl-2 inhibitor)	MTD, RP2D
NCT02520778	1	50	Osimertinib and navitoclax (Bcl-2 inhibitor)	Incidence of toxicity
NCT02917993	1/2	59	Osimertinib + itacitinib (JAK1 inhibitor)	AEs, DLT and ORR
NCT03455829	1/2	30	Osimertinib + lerociclib (CDK4/6 inhibitor)	DLT, RP2D, AEs and PFS
NCT04545710	2	18	Osimertinib + abemaciclib (CDK4/6 inhibitor)	PFS
NCT03944772 (ORCHARD)	2	150	Osimertinib + gefitinib; Osimertinib + necitumumab (anti-EGFR mAb); Osimertinib + alectinib (ALK inhibitor); Osimertinib + selpercatinib (RET inhibitor); Osimertinib + savolitinib	ORR
NCT02143466 (TATTON)	1	344	Osimertinib + selumetinib (MEK inhibitor); Osimertinib + savolitinib; Osimertinib + durvalumab (PD-L1 mAb)	Number of participants with AEs
Chemotherapy				
NCT04765059 (COMPEL)	3	204	Osimertinib + chemotherapy; Placebo + chemotherapy	PFS
NCT04769388 (FLAME)	2	150	Osimertinib monotherapy; Osimertinib + chemotherapy	PFS
NCT04035486 (FLAURA2)	3	587	Osimertinib; Osimertinib + pemetrexed/cisplatin; Osimertinib + pemetrexed/carboplatin	-PFS
NCT04695925 (TOP)	3	291	Osimertinib; Osimertinib + pemetrexed/carboplatin	PFS
NCT03567642	1	20	Osimertinib + platinum/etoposide	MTD
Immunotherapy				
NCT04099836	2	39	Atezolizumab + bevacizumab	ORR
NCT02496663	1	100	Osimertinib + necitumumab (anti-EGFR antibody)	MTD and incidence of toxicity
NCT04285671	1/2	26	Osimertinib + necitumumab + trastuzumab	RP2D, AEs, ORR
NCT03133546 (BOOSTER)	2	155	Osimertinib + bevacizumab; Osimertinib	PFS
NCT04181060	3	300	Osimertinib + bevacizumab; Osimertinib	PFS
NCT03909334	2	150	Osimertinib + ramucirumab; Osimertinib	PFS
NCT03784599 (TRAEMOS)	2	58	Osimertinib + trastuzumab emtansine (HER2 ADC)	ORR
NCT03505710 (DESTINY-Lung01)	2	181	Trastuzumab deruxtecan (HER2 ADC)	ORR
NCT04644237 (DESTINY-Lung02)	2	150	Trastuzumab deruxtecan	ORR
NCT04619004 (HERTHENA-Lung01)	2	420	Patritumab deruxtecan (HER3 ADC)	ORR
NCT05338970 (HERTHENA-Lung02)	3	560	Patritumab deruxtecan; Platinum + pemetrexed	PFS
NCT04676477	1	252	Osimertinib + patritumab deruxtecan	DLT, AEs, ORR
NCT03539536	2	270	Telisotuzumab vedotin (ABBV-399, a MET ADC)	ORR, AEs
NCT02099058	1	260	ABBV-399; ABBV-399 + nivolumab; ABBV-399 + erlotinib; ABBV-399 + osimertinib	AEs, RP2D
NCT04484142 (TROPION-Lung05)	2	137	Datopotamab deruxtecan (TROP2 ADC)	ORR
NCT04526691 (TROPION-Lung02)	1	120	Datopotamab deruxtecan + pembrolizumab; Datopotamab deruxtecan + pembrolizumab + platinum	DLTs
NCT04487080 (MARIPOSA)	3	1074	Amivantamab + lazertinib; Osimertinib; Lazertinib	-PFS
Chemoimmunotherapy				
NCT03515837 (KEYNOTE789)	3	492	Platinum + pemetrexed; Platinum + pemetrexed + pembrolizumab	PFS and OS
NCT02864251 (CheckMate722)	3	274	Platinum + pemetrexed; Platinum + pemetrexed + nivolumab; Ipilimumab + nivolumab	PFS
NCT03786692	2	117	Atezolizumab + bevacizumab + carboplatin + pemetrexed; Bevacizumab + carboplatin + pemetrexed	PFS
NCT04147351	2	22	Atezolizumab + bevacizumab + platinum + pemetrexed	ORR
NCT03991403	3	228	Atezolizumab + bevacizumab + carboplatin + paclitaxel; Platinum + pemetrexed	PFS
NCT02609776 (CHRYSALIS)	1	780	Amivantamab (EGFR/MET BsAb); Amivantamab + lazertinib (EGFR inhibitor); Amivantamab + lazertinib + carboplatin/pemetrexed	DLT, AEs, ORR, DOR
NCT05299125 (AMIGO-1)	2	49	Amivantamab + lazertinib + carboplatin/pemetrexed	PFS
NCT04988295 (MARIPOSA-2)	3	500	Amivantamab + lazertinib + carboplatin/pemetrexed; Carboplatin/pemetrexed; Amivantamab + carboplatin/pemetrexed;	PFS

*ADC* antibody–drug conjugate, *AEs* adverse events, *BsAb* bispecific antibody, *DLT* dose-limiting toxicities, *DOR* duration of response, *EGFR* epidermal growth factor receptor, *mAb* monoclonal antibody, *MTD* maximum tolerated dose, *NSCLC* non-small cell lung cancer, *OD* optimal dosage, *ORR* overall response rate, *OS* overall survival, *PFS* progression-free survival, *Pts* patients, *RP2D* recommended phase 2 dose, *TKI* tyrosine kinase inhibitor

Overall, osimertinib combined with a MET inhibitor is a feasible choice for EGFR-mutated NSCLC patients with resistance to osimertinib caused by MET amplification.

##### Osimertinib plus other tyrosine kinase receptor inhibitors

HER2 amplification is another important off-target resistance mechanism of osimertinib, and a therapeutic regimen targeting HER2 will be discussed in the section on antibody‒drug conjugates in immunotherapy. In this section, we mainly discuss other tyrosine kinase receptors. Fibroblast growth factor receptors (FGFRs), consisting of FGFR1, FGFR2, FGFR3 and FGFR4, are implicated in the progression of a range of cancers; in particular, FGFR1 shows oncogenic traits in lung cancer [137]. Alvaro et al. reported that FGFR1 cooperated with EGFR to enhance the carcinogenic properties of FGFR1 in models of lung adenocarcinoma, and at the clinical level, high FGFR1 expression predicted stronger resistance to first-generation EGFR-TKIs in lung adenocarcinoma patients [137]. Interestingly, concomitant inhibition of FGFR1 and EGFR showed synergistic action on tumor regression in EGFR-mutated, FGFR1-overexpressing xenograft models. In addition, Terp et al. demonstrated that a combination regimen of an FGFR inhibitor and an AKT inhibitor was more effective in overcoming EGFR-TKI resistance than an FGFR inhibitor plus EGFR-TKI, suggesting that inhibition of the FGFR1-AKT pathway might be a promising strategy in EGFR-TKI-resistant NSCLC bearing FGFR1 overexpression [138].

Similar to FGFR1, insulin-like growth factor 2 (IGF2) autocrine-mediated insulin-like growth factor 1 receptor (IGF1R) pathway activation confers acquired resistance to osimertinib [84, 139]. An in vivo study showed that inhibition of IGF1R via linsitinib (an IGF1R inhibitor) or knockdown of IGF1R enabled NSCLC cells to regain sensitivity to osimertinib. Finally, AXL is a tyrosine kinase receptor and high expression of AXL contributes to sustained cell survival and fosters the occurrence of osimertinib-resistant cells. The AXL inhibitor NSP1034 delayed tumor growth when combined with osimertinib [82]. Additionally, ONO-7475 also conferred AXL-overexpressing, EGFR-mutated NSCLC cells sensitivity to osimertinib. Consistent with NSP1034, ONO-7475 together with osimertinib markedly suppressed tumor regrowth [140]. Because of the promising efficacy of the AXL inhibitor in preclinical studies, a clinical trial (NCT03255083) is investigating the safety and tolerability of DS-1205c (an orally administered AXL inhibitor) combined with osimertinib among EGFR-mutated advanced NSCLC subjects.

Currently, however, except for some preclinical investigations, no case reports or clinical trials have demonstrated the effectiveness of osimertinib combined with corresponding TKIs in NSCLC patients with osimertinib resistance mediated by these tyrosine kinase receptors. In the face of this issue, standard platinum-based doublet chemotherapy is potentially a practical therapeutic strategy.

##### Combining osimertinib with inhibitors of oncogenic fusions

As oncogenic drivers, oncogenic fusions are responsible for acquired resistance to osimertinib by activating bypass signaling pathways, which are observed in 1–10% of cases [63, 64].

An in vitro study showed that CCDC6-RET fusion conferred osimertinib resistance but resensitized osimertinib plusBLU-667 (RET inhibitor) by suppressing both ERK and AKT phosphorylation. On the basis of this preclinical study, two EGFR-mutated, RET-fused NSCLC patients benefited from the combination therapy of osimertinib and BLU-667 with a tolerable profile and rapid radiographic response [89]. Moreover, the combination therapy of osimertinib plus cabozantinib (a multitargeted receptor TKI) was applied to treat a patient with CCNYL2-RET fusion, with a PFS of 5 months in a retrospective analysis [141]. Zhang et al. reported that the combination of crizotinib and osimertinib was effective in overcoming GOPC-ROS1 rearrangement-mediated osimertinib resistance [88]. Similarly, osimertinib plus crizotinib also achieved partial response in two patients harboring MET fusion [90, 142]. Finally, EML4-ALK fusion could be successfully managed with osimertinib plus alectinib (ALK inhibitor) or crizotinib [85, 87].

Combining osimertinib with an inhibitor targeting the corresponding oncogenic fusion appeared to be a plausible therapeutic strategy for oncogenic fusion-induced resistance to osimertinib based on the abovementioned results. However, although combination therapy of osimertinib and trametinib (MEK inhibitor) successfully inhibited the proliferation of NSCLC cell lines harboring BRAF fusions in a synergistic manner, a patient with AGK-BRAF fusion had difficulty tolerating such a combination regimen and ultimately discontinued treatment, owing to overlapped toxicity of inhibitors of EGFR and MAPK pathway [143, 144]. This phenomenon indicates that not all such combination strategies are tolerable and feasible, which must attract the attention of physicians. In addition, oncogenic fusions often coexist with other genetic alterations or resistance mechanisms, contributing to increasing the difficulty of applying such combination regimens [141, 145].

Collectively, oncogenic fusions are relatively rare but targetable mechanisms of resistance to osimertinib. In regard to progression on osimertinib treatment, fusion detection should be taken into consideration, and combining osimertinib with an inhibitor targeting oncogenic fusion is warranted to perform further studies to assess the applicability of different combination regimens.

##### Osimertinib plus inhibitors of downstream signaling pathways

EGFR downstream signaling pathways consist of the PI3K pathway, MAPK pathway and JAK/STAT pathway (Fig. 2b). NRAS, KRAS and BRAF mutations are the predominant alterations of the MAPK pathway that lead to osimertinib resistance [91, 146–148]. Several preclinical studies have demonstrated that inhibition of MEK/ERK potently overcame acquired resistance to osimertinib [149, 150]. Combination treatment with osimertinib and selumetinib (MEK inhibitor) hindered resistance development and gave rise to tumor regression in vitro and in vivo [148]. Similarly, osimertinib combined with trametinib (another MEK inhibitor) also overcame KRAS mutation-induced resistance to osimertinib in vitro and in vivo [151, 152]. Preliminary results obtained from the TATTON study revealed that osimertinib combined with selumetinib exhibited an ORR of 42% and a median DOR of 16.6 months with a manageable toxicity profile in EGFR-mutated NSCLC patients whose disease progressed on a previous EGFR-TKI [134]. Furthermore, the safety and effectiveness of osimertinib plus selumetinib as first-line treatment are also being investigated in a phase II clinical study (NCT03392246). Therefore, dual inhibition of EGFR and MEK might be a promising approach against RAS mutation-mediated osimertinib resistance in EGFR-mutated NSCLC patients. In addition, osimertinib, dabrafenib (BRAF inhibitor) and trametinib three-drug combination therapy reached a PFS of 7.4 months and 13.4 months in two NSCLC patients with BRAF V600E mutation-mediated resistance to osimertinib, respectively [92, 153]. Several other case reports also demonstrated the clinical response of this three-drug regimen to patients with BRAF V600E, which together indicate the feasibility of such a therapeutic strategy in the BRAF V600E mutation setting [154–156].

In terms of the PI3K/AKT signaling pathway, PIK3CA encodes the p110α protein, which is a catalytic subunit of PI3K, and PIK3CA mutations contribute to persistent activation of the PI3K/AKT pathway, promoting the development of resistance. Zhang et al. reported that inhibition or knockout of PI3K 110α and 110β reversed multidrug resistance induced by ATP-binding cassette transporter and AKT activation in NSCLC cells [157]. Moreover, mTOR is a downstream molecule of the PI3K/AKT pathway, and dactolisib (dual PI3K and mTOR inhibitor) combined with osimertinib overcame resistance to osimertinib both in vivo and in vitro [158, 159]. Importantly, osimertinib combined with sapanisertib (mTOR inhibitor) is being investigated in a phase II study (NCT02503722) for EGFR-mutated NSCLC patients who are resistant to previous first-line osimertinib treatment. Recently, Han et al. reported that osimertinib plus aspirin increased Bim expression and resulted in robust antiproliferative and proapoptotic effects in vitro and in vivo by inhibiting phosphorylation of the AKT/FoxO3a signaling pathway [160]. A retrospective analysis of forty-five NSCLC patients indicated that osimertinib plus aspirin yielded a significantly longer median PFS than osimertinib alone (15.3 versus 9.3 months, *P* = 0.023) [160]. In view of these previous results, an observational trial (NCT03532698) is being conducted to assess the feasibility of a combined regimen with osimertinib plus aspirin in patients with PI3K pathway alterations.

Finally, JAK/STAT pathway activation is related to a wide spectrum of diseases; specifically, elevated phosphorylation of STAT3 has been found to be associated with poor response to drugs targeting AKT and MAPK signaling pathways [161]. Golidocitinib (AZD4205, a highly selective JAK1 inhibitor) was reported to enhance antitumor activity when combined with osimertinib in a NSCLC xenograft model [161]. In addition, osimertinib combined with itacitinib (JAK1 inhibitor) is under investigation in the clinical trial (NCT02917993).

Given the crosstalk among different signaling pathways, targeting a single pathway could not effectively overcome osimertinib resistance mediated by alterations in signaling pathways. Concomitant inhibition of several pathways could be a more proper strategy; however, there may be considerable overlap in the toxicity profile of multiple inhibitors, and inhibition of several signaling pathways possibly interfere with normal physiological function. The risk–benefit of such a regimen deserves further investigation.

##### Combination of osimertinib and CDK4/6 inhibitors

Cyclin-dependent kinase (CDK) inhibitors can inhibit the transition from G1 to S phase of the cell cycle by decreasing CDK-induced phosphorylation of downstream Rb. CDK4/6 inhibitors (e.g., palbociclib and abemaciclib) combined with osimertinib markedly increased the percentage of G1 phase cells and blocked proliferation of osimertinib-resistant cells, owing to the reduced level of Rb phosphorylation by inhibiting the function of CDK4/6 [162, 163]. Moreover, it has been reported that CDK7 inhibitors are a potential treatment option to cope with EMT-mediated resistance to osimertinib in NSCLC patients [164]. Two clinical trials are underway to investigate the potential clinical benefit of CDK4/6 inhibitors (lerociclib or abemaciclib) together with osimertinib in EGFR mutation-positive metastatic NSCLC patients who had progressed on osimertinib treatment in NCT03455829 and NCT04545710, respectively.

In this section of targeted therapy, the predominant therapeutic strategies are biomarker-driven approaches, that is, osimertinib combined with inhibitors of the corresponding targets. The ORCHARD study adopted a biomarker-driven strategy to design different combination schemes to evaluate the efficacy and safety of osimertinib combined with other inhibitors in NSCLC patients with targetable resistance mechanisms. [165] (Table 3). Although a portion of biomarker-driven strategies are being evaluated in clinical trials and have been reported in a number of case reports, a substantial portion of these strategies are restricted to the preclinical stage. The potential targeted therapeutic strategies for EGFR-independent mechanisms are shown in Fig. 4b.

### Chemotherapy

Although a variety of on-target and off-target mechanisms of resistance to osimertinib have been identified, 30–50% of resistance mechanisms remain unknown. Targeted therapy is not feasible for patients without targetable resistance mechanisms, whereas platinum-based doublet chemotherapy is the practical approach, especially for those with SCLC transformation. The results from a retrospective study suggested that chemotherapy exhibited a tendency toward better survival than a nonchemotherapy regimen in advanced NSCLC patients who progressed on the therapy of osimertinib. The median OS was 25.0 versus 11.8 months, while no significant difference was observed in terms of PFS [166]. SCLC transformation is an important mechanism that confers resistance to osimertinib, which arises in 2%-15% of cases [63, 64]. Analogous to de novo SCLC, a platinum-based doublet chemotherapy regimen, especially platinum-etoposide combination therapy, is routinely recommended to treat patients with SCLC transformation after osimertinib treatment. A case report revealed that cisplatin-etoposide showed an unexpectedly favorable response with a PFS of 7.7 months in an SCLC-transformed patient [167]. In addition, a retrospective analysis showed that platinum-etoposide achieved a clinical response rate of 54%, median PFS of 3.4 months and median OS of 10.9 months in SCLC-transformed patients, while no responses were observed among those who received immune checkpoint inhibitors [168].

The continuing administration of EGFR-TKIs in the course of chemotherapy remains controversial. The IMPRESS phase III randomized trial demonstrated that compared with cisplatin and pemetrexed alone, no difference was observed in PFS (5.4 vs. 5.4 months) but worse OS (13.4 vs. 19.5 months) occurred in patients who received continuation of gefitinib after progression on first-line gefitinib treatment [169, 170]. Conversely, the first-line gefitinib combined with carboplatin plus pemetrexed achieved longer PFS (20.9 versus 11.9 months) and OS (49.0 vs. 38.5 months) than gefitinib alone in the NEJ009 phase III trial [171, 172]. Combination therapy with gefitinib and chemotherapy generates opposite results in the different treatment settings, which probably reflects gene alterations in NSCLC patients after receiving EGFR-TKIs.

However, clinical data on whether to continue osimertinib when initiating chemotherapy after progression on front-line osimertinib therapy are limited. Recently, a randomized phase II clinical study explored the effect of osimertinib in combination with carboplatin-pemetrexed compared to osimertinib monotherapy among EGFR-mutated NSCLC patients withT790M in the second-line setting. The median PFS was 15.8 months versus 14.6 months, and the ORR was 71.4% versus 53.6% in the osimertinib monotherapy group and combination therapy group, respectively [173]. Similar to gefitinib, combination therapy with osimertinib plus chemotherapy did not improve survival in the second-line setting. To further confirm these results, the COMPEL and FLAME studies are ongoing to compare osimertinib plus chemotherapy with osimertinib monotherapy in EGFR-mutant NSCLC. Moreover, first-line osimertinib combined with platinum-based chemotherapy versus osimertinib alone is also being assessed in the FLAURA2 phase III trial (Table 3). These clinical trials will provide insight into whether combination therapy of osimertinib and chemotherapy is a feasible therapeutic strategy for patients with osimertinib resistance who are unsuitable for the aforementioned targeted therapy. Finally, although there is a lack of sufficient clinical evidence, in consideration of the favorable efficacy against CNS metastases and the well-tolerated safety profile of osimertinib, continuing osimertinib when initiating second-line chemotherapy is considered a practicable therapeutic regimen for NSCLC patients suffering baseline brain metastases. Interestingly, such a scheme has been successfully applied in a patient with meningeal carcinomatosis whose extracranial disease progressed on osimertinib [174].

### Immunotherapy

#### Immune checkpoint inhibitors

Platinum-based chemotherapy is the first-line therapy for advanced NSCLC patients who lack targetable mutations, but among those with PD-L1 expression ≥ 50%, immune checkpoint inhibitor (ICI) is a preferential option. Compared with chemotherapy, first-line pembrolizumab achieved a significantly longer PFS of 10.3 versus 6.0 months and a higher ORR of 44.8% versus 27.8%, and second-line pembrolizumab also prolonged OS (14.9 months in the pembrolizumab 2 mg/kg group vs. 8.2 months in the docetaxel group and 17.3 months in the pembrolizumab 10 mg/kg group vs. 8.2 months in the docetaxel group, respectively) in PD-L1-positive, advanced NSCLC patients without EGFR mutations or ALK rearrangement [175, 176]. The exciting results derived from these phase III trials indicated that ICIs contributed to improving the survival of NSCLC patients without targetable mutations; however, ICI monotherapy exerts limited benefit in those with EGFR mutations. A systematic review suggested that ICIs were associated with prolonged OS (HR, 0.69; 95% CI, 0.63–0.75; *P* < 0.001) in individuals harboring wild-type EGFR but not in the EGFR mutation subgroup (HR, 1.11; 95% CI, 0.80–1.53; *P* = 0.54) compared to docetaxel [177]. Similarly, the results from the IMMUNOTARGET registry showed that ICI monotherapy yielded a short median PFS of 2.1 months and a low ORR of 12% among NSCLC patients harboring EGFR alterations [178]. Furthermore, pembrolizumab was found to provide limited effect in EGFR-mutant advanced NSCLC population who never received TKI treatment, even among those with PD-L1 expression more than 50%, indicating ICI monotherapy was not a suitable therapeutic choice for this group of patients [179]. It is worth noting that ICIs increase the incidence of hyperprogressive diseases (HPD) that are related to worse survival, imposing restrictions on the clinical application of ICI monotherapy in NSCLC with targetable mutations [180, 181].

Given that ICI monotherapy showed little clinical benefit in EGFR-mutated NSCLC patients, combination strategies have been investigated. The phase III CAURAL trial (NCT02454933) aimed to compare osimertinib plus durvalumab with osimertinib alone in EGFR-mutant advanced NSCLC patients whose disease progressed on EGFR-TKIs [182]. Nevertheless, the investigational recruitment of the CAURAL trial was discontinued prematurely due to an increased incidence of interstitial lung disease-like adverse events in the osimertinib plus durvalumab cohort from the TATTON study [134]. Notably, the application sequence of ICIs and osimertinib was associated with adverse events (AEs). Schoenfeld et al. reported that sequential ICI followed by osimertinib were relevant to severe immune-associated AEs, especially in those who recently received ICIs [183]. Conversely, no identified severe immune-related AEs were observed in patients administered osimertinib followed by ICI treatment. Thus, caution should be taken when osimertinib is used in patients recently treated with ICIs.

Furthermore, the combined effects of immunotherapy and chemotherapy (that is, chemoimmunotherapy) have also been investigated in different conditions. In contrast with chemotherapy alone, sintilimab, an anti-PD-1 antibody, combined with chemotherapy contributed to better clinical benefit, with PFS of 8.9 versus 5.0 months and ORR of 51.9% versus 29.8% in the first-line setting for the treatment of metastatic in NSCLC patients without EGFR or ALK mutations [184, 185]. Similarly, atezolizumab or pembrolizumab plus platinum-based chemotherapy have remarkably prolonged survival compared with chemotherapy alone in clinical trials (IMpower131, KEYNOTE-189 and KEYNOTE-407) [186–188]. In addition, nivolumab combined with bevacizumab, carboplatin and paclitaxel also achieved longer PFS (12.1 versus 8.1 months) than placebo combinations in the first-line treatment of NSCLC without targetable mutations [189]. These results support the role of chemoimmunotherapy as a first-line regimen for metastatic NSCLC patients without EGFR mutations. Nevertheless, for those with EGFR mutations, the survival benefit of chemoimmunotherapy is not exactly clear because EGFR-mutant patients were completely excluded from most clinical trials with immunotherapy, except for a few trials. In the PROLUNG trial, the combination of pembrolizumab plus docetaxel substantially improved ORR (58.3% vs. 23.1%) and PFS (6.8 vs. 3.5 months) compared with docetaxel alone in patients with EGFR mutations who progressed on platinum-based chemotherapy [190]. The Impower130 study showed that no survival benefit was observed in the EGFR-mutant subgroup administered atezolizumab and carboplatin/nab-paclitaxel (ACP regimen) compared to chemotherapy alone; however, the IMpower150 trial demonstrated that the addition of atezolizumab and bevacizumab to carboplatin/paclitaxel (ABCP regimen) significantly improved PFS and OS in EGFR-mutated NSCLC patients in contrast with bevacizumab plus carboplatin/paclitaxel (BCP regimen) [191–193]. Similarly, another clinical trial showed that combination treatment with atezolizumab, bevacizumab, carboplatin and pemetrexed also achieved promising efficacy in metastatic EGFR-mutated NSCLC patients after EGFR-TKI failure [194]. Recently, interim results from the ORIENT-31 phase III trial (NCT03802240) indicated that PFS was significantly longer (6.9 months vs. 4.3 months) in the sintilimab plus IBI305 (bevacizumab biosimilar) plus cisplatin and pemetrexed group (SBCP regimen) than in the chemotherapy alone group in EGFR-mutated NSCLC patients who progressed on previous EGFR-TKIs therapy [195]. Taken together, these results suggest a synergistic effect of ICIs and antiangiogenic drugs on chemotherapy for the treatment of NSCLC patients in EGFR-mutated settings. To further confirm the efficacy of ICIs plus antiangiogenic agents with or without chemotherapy on patients harboring EGFR mutations in larger cohorts, different combination regimens are under investigation (Table 3).

##### Anti-VEGF/VEGFR antibodies

It has been reported that the VEGF pathway is implicated in EGFR-TKI resistance and that the addition of bevacizumab to erlotinib substantially improved PFS (17.9 months vs. 11.2 months) compared with erlotinib alone in EGFR-mutant NSCLC patients [196, 197]. Therefore, a combination regimen of osimertinib with antiangiogenic drugs has also been investigated in both first-line and second-line settings. In a phase I/II single-arm trial, combination therapy of osimertinib with bevacizumab as first-line treatment reached an ORR of 80% and a median PFS of 19 months for patients with EGFR-mutant lung cancers [198]. However, the phase II WJOG9717L study failed to exhibit improved PFS (22.1 months vs. 20.2 months) in the osimertinib plus bevacizumab group compared with osimertinib alone in the first-line setting [199]. It is disappointing that in the second-line setting, osimertinib plus bevacizumab also failed to extend PFS in EGFR-mutant NSCLC patients [200, 201]. The discrepancy between the survival benefit of erlotinib plus bevacizumab and osimertinib plus bevacizumab possibly reflects tumor heterogeneity caused by different EGFR-TKIs. In addition, ramucirumab (anti-VEGFR2 antibody) plus osimertinib demonstrated encouraging safety and antitumor activity among EGFR-mutant NSCLC patients in a phase I study (NCT02789345) [202]. Additional studies on the combination treatment of osimertinib with bevacizumab or ramucirumab are shown in Table 3.

##### Anti-EGFR antibodies

In addition to small molecule inhibitors targeting the intracellular tyrosine kinase domain of EGFR (i.e., EGFR-TKIs), anti-EGFR antibodies that block the extracellular ligand-binding region of EGFR have also been developed. Cetuximab, an anti-EGFR monoclonal antibody, when combined with osimertinib and trastuzumab (anti-HER2 antibody), was able to prevent resistance to osimertinib in vitro and in vivo [203]. In addition, cetuximab plus osimertinib demonstrated a promising effect on an NSCLC patient with EGFR exon 20 insertion mutation [204]. Moreover, another anti-EGFR monoclonal antibody, necitumumab, when used in combination with chemotherapy as the first-line therapy, achieved a longer OS (11.5 months vs. 9.9 months) than chemotherapy alone among advanced squamous NSCLC patients [205]. This study contributed to the approval of necitumumab plus gemcitabine and cisplatin for the first-line treatment of metastatic squamous NSCLC [206]. Several clinical trials (NCT03944772, NCT04285671 and NCT02496663) are evaluating the efficacy of necitumumab plus osimertinib in NSCLC with progression on EGFR-TKIs, including osimertinib.

##### Bispecific antibody

Compared to other monoclonal antibodies (mAbs) that target single molecules, bispecific antibodies (BsAbs) are designed to target two specific molecules simultaneously. Considering the multiple gene alterations and heterogeneity of tumors, BsAbs may exhibit better antitumor activity. Amivantamab (JNJ-61186372, JNJ-6372, JNJ-372) is an engineered recombinant BsAb that targets EGFR and MET [207]. This bispecific EGFR-MET antibody inhibits ligand-induced activation of EGFR and MET and further blocks phosphorylation of downstream signaling molecules. Amivantamab presented preclinical activity against EGFR-mutated NSCLC cells with EGFR-TKIs resistance; importantly, the antitumor efficacy of amivantamab was also observed in two patients with exon20ins, which supports further clinical development [208, 209].

The CHRYSALIS phase I study revealed that amivantamab yielded an ORR of 40%, PFS of 8.3 months and OS of 22.8 months with a tolerable safety profile in exon20ins-driven NSCLC patients after progression on platinum-based chemotherapy [210]. On the basis of encouraging results from the CHRYSALIS, amivantamab received its first approval by the FDA for the treatment of locally advanced or metastatic NSCLC with exon20ins who progressed on platinum-based chemotherapy on 21 May, 2021 [211]. Moreover, the CHRYSALIS study also demonstrated that amivantamab combined with lazertinib showed antitumor activity in the osimertinib-resistant setting, and the MARIPOSA study (NCT04487080) is ongoing to compare the efficacy and safety of amivantamab plus lazertinib with osimertinib alone as first-line treatment for EGFR-mutant NSCLC [212]. More importantly, similar to the ABCP regimen, the MARIPOSA-2 trial (NCT04988295) is designed to assess the efficacy of the ALCP regimen (amivantamab, lazertinib, carboplatin and pemetrexed), ACP regimen (amivantamab, carboplatin and pemetrexed) and CP regimen (carboplatin and pemetrexed) in subjects with EGFR-mutant NSCLC after osimertinib failure. This study will stand a good chance of offering a novel therapeutic strategy to cope with the intractable issue of osimertinib resistance.

##### Antibody‒drug conjugates

**Fig. 5:**
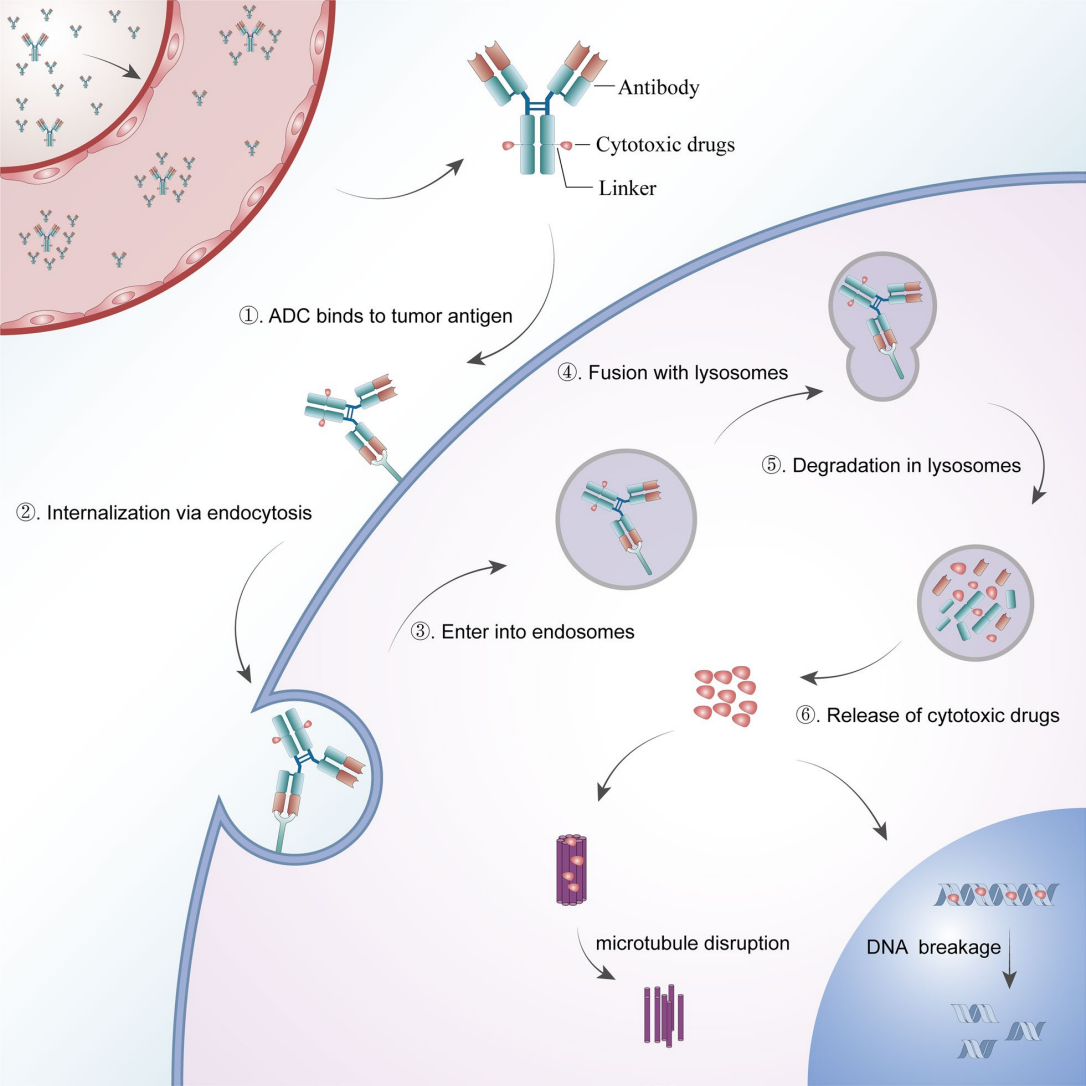
The core mechanism of action of antibody–drug conjugates (ADCs). ADCs contain three parts: monoclonal antibody targeting tumor antigen, cytotoxic drugs and a linker that combines antibody with drugs. ADC binds to specific antigens expressed on tumor cells, followed by internalization via endocytosis. The ADCs enter endosomes and subsequently degrade in lysosomes, resulting in the release of cytotoxic drugs. Cytotoxic drugs contribute to DNA breakage and microtubule disruption, ultimately inducing the death of tumor cells

Antibody‒drug conjugates (ADCs) are novel compounds that combine humanized monoclonal antibodies and cytotoxic drugs by a linker to exert antitumor effects by targeting neoplastic antigens on tumor cells. ADCs integrate the high specificity of antibodies and the high activity of cytotoxic drugs, which yield more potent efficacy against tumors with less toxicity. The mechanism of action of ADCs is presented in Fig. 5. Currently, over one hundred ADCs are under clinical investigation, and more than a dozen ADCs have received approval for the treatment of hematological malignancies and solid tumors; in particular, T-DM1, a HER2-targeting ADC, was the first approved ADC in solid tumors for HER2-positive metastatic breast cancer [213–215].

Considering the promising antitumor effect of ADCs, ADCs have also been investigated in NSCLC. HER2-targeted regimens are firstly used in HER2-positive breast and gastric cancers, and two HER2-targeting ADCs, trastuzumab emtansine (T-DM1) and trastuzumab deruxtecan (T-DXd, DS-8201), have been approved for the treatment of HER2-positive breast cancer and gastric cancers. However, T-DM1 showed limited effect for HER2-positive NSCLC patients, even in those with HER2-overexpressing NSCLC [216, 217]. In contrast to T-DM1, in the DESTINY-Lung01 study, T-DXd reached a median PFS of 8.2 months and a median OS of 17.8 months with acceptable toxic effects in previously treated patients with HER2-mutated NSCLC patients [218]. Excitingly, based on the encouraging results of the DESTINY-Lung02 phase II trial, the FDA granted accelerated approval to T-DXd for NSCLC patients harboring HER2 mutations who received systemic therapy on August 11, 2022 [219]. In a preclinical study, concomitant administration of osimertinib and T-DM1 exerted an additive effect on the inhibition of NSCLC cells and xenograft models [220]. Furthermore, the TRAEMOS trial (NCT03784599) is investigating the clinical efficacy of such a combination regimen among advanced EGFR-mutant NSCLC patients with HER2 resistance who have progressed on EGFR-TKIs.

HER3 (ErbB3) is often expressed in EGFR-mutated NSCLC, but it has not been identified to confer resistance to EGFR-TKIs. The HER3-targeting ADC patritumab deruxtecan (HER3-DXd, U3-1402) demonstrated clinical activity against EGFR-TKI-resistant NSCLC in the HERTHENA-Lung01 study, which provides a strategy to treat osimertinib-resistant NSCLC [221]. Thus, the antitumor activity of HER3-DXd alone or combined with osimertinib in NSCLC with progression on EGFR-TKIs treatment is being assessed in the clinical trials HERTHENA-Lung02 and NCT04676477, respectively.

Telisotuzumab vedotin (ABBV-399) is a MET-targeting ADC that showed antitumor activity in c-Met-positive NSCLC patients with a favorable safety profile in two phase I studies [222, 223]. Similar to HER3-DXd, ABBV-399 monotherapy and in combination with other drugs, including osimertinib, are also being investigated in several ongoing trials (Table 3).

In addition, trophoblast cell surface antigen 2 (TROP2) is an intracellular calcium signaling transducer that is highly expressed in some solid tumors, including NSCLC. Datopotamab deruxtecan (Dato-DXd, DS-1062a), a TROP2-targeting ADC showed satisfactory results for the treatment of NSCLC in a phase I study, and this ADC is being further evaluated in the TROPION-Lung 02 and TROPION-Lung 05 trials [224]. Another TROP2-targeting ADC, sacituzumab govitecan (IMMU-132), also induced durable responses in pretreated metastatic NSCLC patients [225]. These aforementioned clinical trials on ADC monotherapy or combinations will help us broaden the spectrum of therapeutic strategies for patients with osimertinib resistance.

**Fig. 6:**
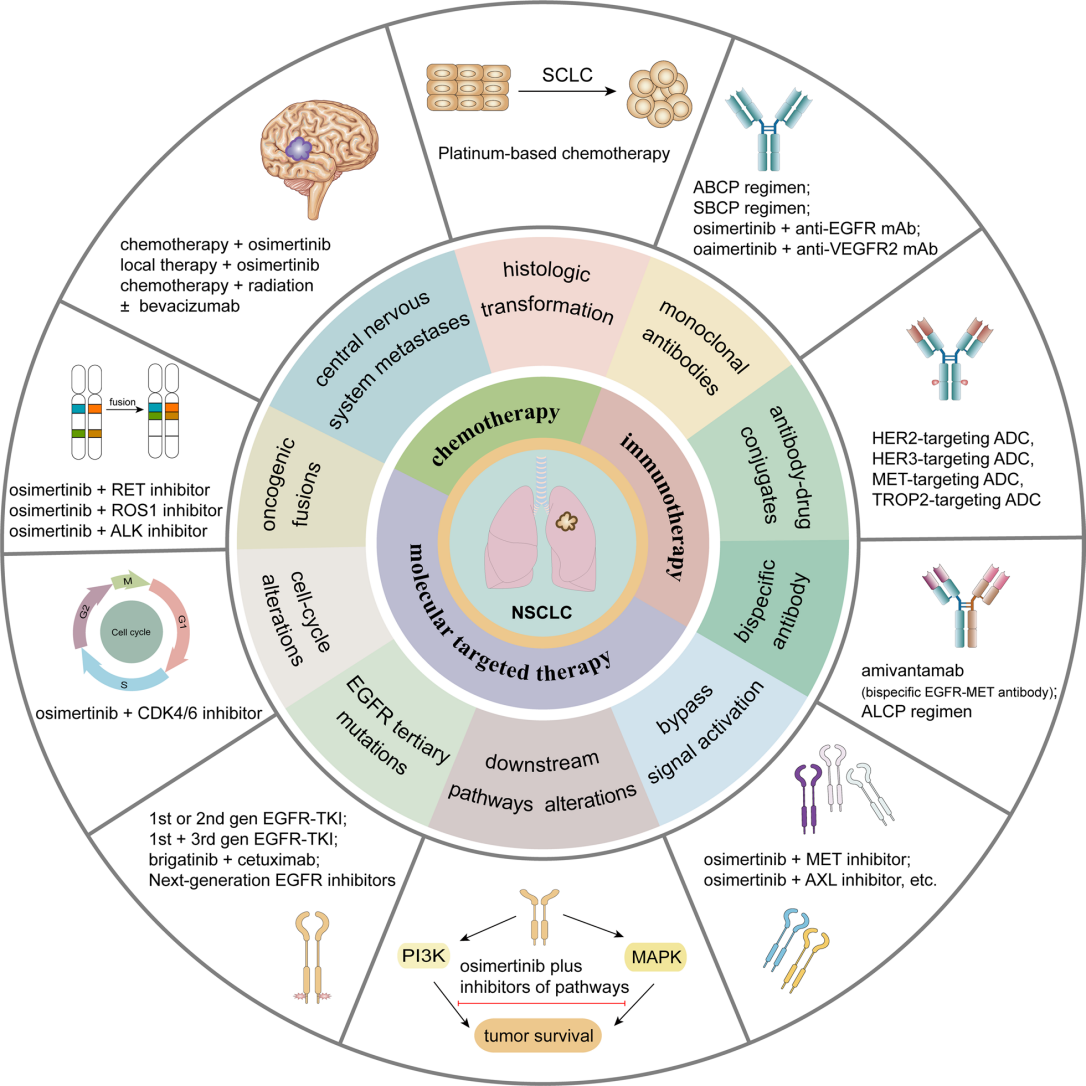
Summary of therapeutic strategies for osimertinib resistance. Therapeutic strategies are comprised of targeted therapy, chemotherapy and immunotherapy; in addition, combination regimens will be considered depending on the individual characteristics of patients

Collectively, various potential therapeutic strategies consisting of targeted therapy, chemotherapy and immunotherapy are summarized in Fig. 6.

## Management of CNS metastases

NSCLC represents the most frequent cause of brain metastases, with approximately 20% of advanced NSCLC patients presenting brain metastases at diagnosis and approximately 30% of NSCLC patients developing brain metastases in the course of their diseases [226]. Brain metastases are associated with decreased quality of life and poor prognosis, posing serious threats to cancer patients. The refractory issues of brain metastases, at least in part, reflect low penetration of effective drugs into intracranial lesions due to the existence of the blood–brain barrier (BBB). However, osimertinib exhibits robust BBB penetration compared to early-generation EGFR-TKIs and chemotherapeutic drugs.

The AURA3 trial was the first study to compare the central nervous system (CNS) efficacy of osimertinib with chemotherapy in T790M-positive advanced NSCLC patients with measurable CNS lesions who progressed on previous EGFR-TKI treatment, with a CNS ORR of 70% versus 31% and a median CNS PFS of 11.7 months versus 5.6 months in osimertinib and chemotherapy cohorts, respectively [227]. Similarly, a subgroup analysis of the FLAURA study showed that compared with standard first-line EGFR-TKIs (gefitinib or erlotinib), osimertinib demonstrated a higher CNS ORR (91% versus 68%) and longer CNS PFS (not reached versus 13.9 months) in patients with CNS metastases [228]. Peled et al. also reported that osimertinib displayed potent intracranial activity against EGFR-mutant lung adenocarcinoma with asymptomatic brain metastases in both previously treated and treatment-naive groups, regardless of T790M status [229]. Importantly, in addition to its effect on existing CNS metastases, osimertinib also plays a protective role in CNS progression. The FLAURA study demonstrated that the probability of CNS progression was lower with osimertinib in contrast to gefitinib or erlotinib, with the development of new brain lesions in 12% and 30% of patients, respectively [228]. Additionally, adjuvant osimertinib presented lower recurrence of CNS-related disease (1% vs. 10%) than placebo and exhibited an 82% risk reduction of CNS disease recurrence in the ADAURA trial [230].

In terms of leptomeningeal metastases, the BLOOM study showed that a high dose (160 mg once daily) of osimertinib achieved an ORR of 62%, median PFS of 8.6 months and median OS of 11 months in EGFR-mutated NSCLC patients with leptomeningeal metastases [231]. Additionally, a retrospective analysis of AURA serial studies reported that a standard dose (80 mg once daily) of osimertinib also showed promising activity against leptomeningeal metastases, with an ORR of 55%, a median PFS of 11.1 months and a median OS of 18.8 months [232]. The clinical efficacy of osimertinib in CNS metastases was also observed in other studies [233, 234]. On the strength of the potent CNS efficacy, osimertinib was approved by the FDA for the first-line treatment of metastatic NSCLC patients with EGFR mutations, especially in those with CNS metastases.

CNS progression is still a huge challenge for the long-term management of NSCLC patients. Osimertinib displays more effective CNS activity than early-generation EGFR-TKIs; however, CNS progression on the therapy of osimertinib is unavoidable. When patients’ CNS lesions progress on osimertinib treatment, it is imperative to identify whether extracranial lesion progression exists. If it occurs, we can take advantage of the aforementioned therapeutic strategies to address this situation; if patients only exhibit intracranial progression on osimertinib, the following measures may be taken into consideration. For those with oligometastatic progression lesions, continued osimertinib plus local treatment, including surgery and stereotactic radiotherapy (SRT), will be a preferential choice [56]; platinum-based chemotherapy followed by whole brain radiotherapy (WBRT) with or without bevacizumab is recommended to treat patients with multifocal CNS progression [235–237]. Finally, as discussed above, a high dose of osimertinib exhibited CNS activity in the BLOOM trial [231]; therefore, osimertinib dose escalation to 160 mg in patients with CNS progression who were administered a standard dose (80 mg) may deserve a try. Notably, the efficacy of osimertinib dose escalation from 80 to 160 mg in EGFR-mutated NSCLC patients with brain metastases has been evaluated in a phase II trial, with an intracranial response rate of 54%, which demonstrated that dose escalation of osimertinib was feasible and possibly offered a therapeutic alternative for such patients [238]. However, the efficacy of this approach should be further evaluated.

## Concluding remarks and future perspectives

EGFR-TKIs are considered the optimal approaches for EGFR-mutated advanced NSCLC patients and have remarkably prolonged survival and improved outcomes of this population. Osimertinib is a novel irreversible third-generation EGFR-TKI selective for both EGFR-sensitized mutations and the T790M resistance mutation, which exhibits remarkable clinical activity against CNS metastases due to greater penetration of the BBB than other EGFR-TKIs. Osimertinib is currently the preferable option for EGFR-mutated NSCLC patients because of its potent efficacy and well-tolerated safety profile. However, similar to early-generation EGFR-TKIs, resistance to osimertinib inevitably develops. A growing number of mechanisms of acquired resistance to osimertinib including on-target and off-target resistance mechanisms have been ascertained. Importantly, novel resistance mechanisms probably emerge on account of selective pressure and clonal evolution in the course of osimertinib treatment. As a consequence, to identify the emerging resistance mechanism as soon as possible, it is practicable to repeat molecular analyses through dynamic monitoring technology using serial noninvasive liquid biopsy [239, 240]. It is worth noting that when histologic transformations are considered resistance mechanisms, tumor tissue biopsy must be performed instead [94]. The application of liquid biopsy and tissue biopsy plays a complementary role in detecting the molecular alterations that confer resistance to osimertinib.

The occurrence of resistance to EGFR-TKIs, including osimertinib, hampers the everlasting effective management of NSCLC individuals with EGFR mutations; thus, the development of medicines to overcome the troublesome challenge is urgent and indispensable. Novel techniques for screening anticancer drugs, for example, research advances in drug sensitivity tests at the single-cell level and future application of conditionally reprogrammed primary tumor cells, will contribute to identifying the optimal drug selection for cancer patients in a personalized manner [241, 242]. Therapeutic strategies that combine small molecular inhibitors, immunotherapy and chemotherapy will provide promising treatment regimens for EGFR-mutated NSCLC patients with osimertinib resistance. Several treatment regimens exhibit potent clinical efficacy, whereas more therapeutic strategies are under clinical investigation. An increasing number of clinical trials examine the efficacy and safety of diverse combination treatments, and we look forward to the exciting results from these trials.

Although a portion of mechanisms of resistance to osimertinib have been determined, other considerable resistance mechanisms remain unknown, and further investigations are imperative to broaden the landscape of osimertinib resistance. Full-scale knowledge of the resistance mechanisms of osimertinib sheds light on the exploration of individualized regimens to withstand osimertinib resistance and achieve precise treatment for the sake of improving quality of life and survival of NSCLC patients.

## Data Availability

Not applicable.

## Abbreviations

ADC: Antibody–drug conjugates
ALK: Anaplastic lymphoma kinase
AKT: Protein kinase B
ATP: Adenosine triphosphate
AXL: AXL receptor tyrosine kinase
BBB: Blood–brain barrier
BRAF: B-Raf proto-oncogene, serine/threonine kinase
CDK: Cyclin-dependent kinase
CNS: Central nervous system
DCR: Disease control rate
EGFR: Epidermal growth factor receptor
EMT: Epithelial-mesenchymal transition
ERK: Extracellular signal regulated kinase
FGF2: Fibroblast growth factor 2
FGFR: Fibroblast growth factor receptors
HER2: Human epidermal growth factor receptor 2
HGF: Hepatocyte growth factor
ICIs: Immune checkpoint inhibitors
IGF1R: Insulin-like growth factor 1 receptor
IGF2: Insulin-like growth factor 2
JAK: Janus kinase
KRAS: Kirsten rat sarcoma viral oncogene homologue
MAPK: Mitogen-activated protein kinase
MET: Mesenchymal-epithelial transition
mTOR: Mammalian target of rapamycin
NRAS: Neuroblastoma rat sarcoma virus oncogene
NSCLC: Non-small cell lung cancer
NGS: Next-generation sequencing
ORR: Objective response rate
OS: Overall survival
PD-1: Programmed cell death protein 1
PD-L1: Programmed cell death ligand protein 1
PFS: Progression-free survival
PI3K: Phosphatidylinositol 3-kinase
RAS: Rat sarcoma viral oncogene homologue
RET: Rearranged during transfection
ROS1: C-ros oncogene
SCLC: Small cell lung cancer
SRT: Stereotactic radiotherapy
STAT3: Signal transducer and activator of transcription 3
T-DM1: Trastuzumab emtansine
TKI: Tyrosine kinase inhibitor
VEGF: Vascular endothelial growth factor
VEGFR: Vascular endothelial growth factor receptor
WBRT: Whole brain radiotherapy
